# Diet-induced remission in chronic enteropathy is associated with altered microbial community structure and synthesis of secondary bile acids

**DOI:** 10.1186/s40168-019-0740-4

**Published:** 2019-08-31

**Authors:** Shuai Wang, Rene Martins, Megan C. Sullivan, Elliot S. Friedman, Ana M. Misic, Ayah El-Fahmawi, Elaine Cristina Pereira De Martinis, Kevin O’Brien, Ying Chen, Charles Bradley, Grace Zhang, Alexander S. F. Berry, Christopher A. Hunter, Robert N. Baldassano, Mark P. Rondeau, Daniel P. Beiting

**Affiliations:** 1 Department of Pathobiology, School of Veterinary Medicine, University of Pennsylvania, Philadelphia, PA 19104 USA; 2 Department of Clinical Sciences and Advanced Medicine, School of Veterinary Medicine, University of Pennsylvania, Philadelphia, PA 19104 USA; 3 Division of Gastroenterology, School of Medicine, University of Pennsylvania, Philadelphia, PA 19104 USA; 4 Faculdade de Ciências Farmacêuticas de Ribeirão Preto, Universidade de São Paulo, Ribeirão Preto, SP Brazil; 5 Department of Pediatric Gastroenterology Hepatology and Nutrition, Children’s Hospital of Philadelphia, Philadelphia, PA 19104 USA

**Keywords:** Chronic enteropathy, Dietary therapy, Microbiome, Bile acids, Canine, Metabolomics, Crohn’s disease

## Abstract

**Background:**

The microbiome has been implicated in the initiation and persistence of inflammatory bowel disease. Despite the fact that diet is one of the most potent modulators of microbiome composition and function and that dietary intervention is the first-line therapy for treating pediatric Crohn’s disease, the relationships between diet-induced remission, enteropathy, and microbiome are poorly understood. Here, we leverage a naturally-occurring canine model of chronic inflammatory enteropathy that exhibits robust remission following nutritional therapy, to perform a longitudinal study that integrates clinical monitoring, 16S rRNA gene amplicon sequencing, metagenomic sequencing, metabolomic profiling, and whole genome sequencing to investigate the relationship between therapeutic diet, microbiome, and disease.

**Results:**

We show that remission induced by a hydrolyzed protein diet is accompanied by alterations in microbial community structure marked by decreased abundance of pathobionts (e.g., *Escherichia coli* and *Clostridium perfringens*), reduced severity of dysbiosis, and increased levels of the secondary bile acids, lithocholic and deoxycholic acid. Physiologic levels of these bile acids inhibited the growth of *E. coli* and *C. perfringens* isolates, in vitro. Metagenomic analysis and whole genome sequencing identified the bile acid producer *Clostridium hiranonis* as elevated after dietary therapy and a likely source of secondary bile acids during remission. When *C. hiranonis* was administered to mice, levels of deoxycholic acid were preserved and pathology associated with DSS colitis was ameliorated. Finally, a closely related bile acid producer, *Clostridium scindens*, was associated with diet-induced remission in human pediatric Crohn’s disease.

**Conclusions:**

These data highlight that remission induced by a hydrolyzed protein diet is associated with improved microbiota structure, an expansion of bile acid-producing clostridia, and increased levels of secondary bile acids. Our observations from clinical studies of exclusive enteral nutrition in human Crohn’s disease, along with our in vitro inhibition assays and in vivo studies in mice, suggest that this may be a conserved response to diet therapy with the potential to ameliorate disease. These findings provide insight into diet-induced remission of gastrointestinal disease and could help guide the rational design of more effective therapeutic diets.

**Electronic supplementary material:**

The online version of this article (10.1186/s40168-019-0740-4) contains supplementary material, which is available to authorized users.

## Background

Human inflammatory bowel disease (IBD), including Crohn’s disease and ulcerative colitis, is a multifactorial and debilitating disease characterized by chronic immune-pathology, disruption of intestinal homeostasis, and altered composition of the gut microbiome (dysbiosis). Several lines of evidence point to resident gut bacteria as important factors in the etiology of IBD. First, disease is often more severe in areas of the intestine with the highest microbial biomass, and antibiotics are frequently used as an adjunct therapy with immunosuppressants or monoclonal antibodies for managing IBD [1, 2]. Second, genome-wide association studies have identified numerous susceptibility loci in genes responsible for recognizing or responding to bacteria [3]. Finally, in some mouse models of colitis, disease can be transferred to naive hosts via fecal transplant [4–6], suggesting a causal role for gut microbes in disease. Collectively, these findings have led to a “two-hit” model for IBD in which both host genetics and microbial factors influence disease presentation, highlighting an opportunity to develop novel microbiome-based treatments for IBD.

Although a range of environmental factors have been shown to influence the microbiome, diet is regarded as one of the most potent modulators of the composition and function of the gut-resident microbial community in healthy humans and other mammals [7, 8], and can act as both a risk factor and a treatment modality for IBD [9, 10]. Epidemiologic data and studies in mice have shown that diets high in fat and/or low in fiber, as well as dietary additives such as emulsifiers, are either risk factors for IBD, or in some cases can directly compromise intestinal barrier function leading to disease [11–13]. Diet can be also leveraged to treat IBD, with perhaps the clearest example of this being the use of exclusive enteral nutrition (EEN) as first-line therapy for pediatric Crohn’s disease [14]. High remission rates (≥ 60%) are observed following EEN, and compared to corticosteroids, EEN achieves better patient growth along with a reduction in biomarkers of disease, such as fecal calprotectin and C-reactive protein [15–18]. Interestingly, EEN has a marked effect on the microbiota community, but the precise nature of this effect has been complicated to discern, with some studies reporting reduced microbiome diversity following EEN therapy [19–21], while others point to relatively unchanged [22, 23] or increased diversity [24].

The relationship between diet, microbiome structure, and enteropathy is complicated to dissect from human subject research where diet is challenging to control, necessitating either retrospective studies in conjunction with extensive food intake surveys [25], controlled feeding studies [26], or focusing on populations with different subsistence practices [27–29]. While mouse models have yielded important insights into the pathophysiology of colitis, they often involve chemical or genetic perturbation, rather than spontaneous disease development. Moreover, the ubiquitous use of autoclaved food and acidified water for mouse husbandry, together with the tendency for cage effects to dominate in mouse microbiome studies, raises concerns about clinical relevance of diet-microbiome studies in murine models of colitis. A growing number of studies suggest that dogs are an ideal animal model in which to study translationally relevant diet-microbiome interactions in the context of intestinal disease. First, as companion animals, dogs share the same environment with humans and spontaneously develop a chronic enteropathy (CE) that clinically resembles some aspects of human IBD, including several shared features of gastrointestinal pathology, responsiveness to similar treatments [30, 31], involving some of the same susceptibility loci [30–32], and shared disease-associated microbial taxa [33–35]. Secondly, a recent metagenomic study produced a catalog of over one million taxonomically and functionally annotated microbial genes from the canine gut and showed that—compared to other mammals, such as the mouse and pig—the microbial environment in dogs most closely resembles that of humans [36]. Thirdly, the canine microbiome was markedly altered by diets differing in protein/carbohydrates in a manner that resembles what has been reported in humans [36]. As treatment with dietary therapy promotes a long-lasting state of remission in over 50% of dogs with CE [37], this animal model provides an opportunity to rigorously investigate the relationship between therapeutic diet, microbiome, and disease resolution.

Despite the fact that the gut microbiome has been implicated in IBD pathogenesis and that diet can be used to manage symptoms of IBD, there is limited insight into how this occurs. In this study, we examined dogs with CE and monitored changes in their fecal microbial community structure and metabolites in response to treatment with a therapeutic hydrolyzed protein diet. By comparing changes over time in dogs with food-responsive chronic enteropathy (FRE), versus animals that failed diet therapy and required subsequent combination therapy, we showed that the hydrolyzed protein diet induces rapid remission that is associated with a structural and functional remodeling of the microbial community in the gut. Notably, we observed that secondary bile acids, likely produced by *C. hiranonis*, are associated with the diet-induced remission of canine CE and exert antibacterial activity against isolates of *E. coli* and *C. perfringens* in vitro, suggesting these metabolites may modulate disease. These findings improve our understanding of how dietary therapy can modulate microbial communities and reduce GI disease.

## Results

### Dietary therapy induces rapid and durable remission

**Fig. 1:**
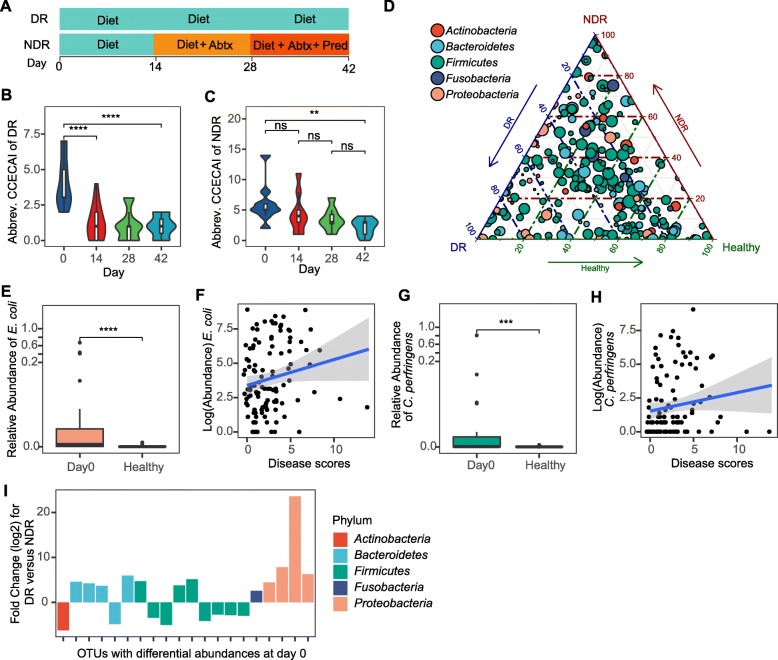
Identification of microbial community profiles associated with treatment outcome in diet therapy. **a** Schematic showing clinical study design for identifying diet-responsive (DR) and non-diet-responsive (NDR) dogs. Antibiotics (Abtx) and prednisone (Pred) treatments are indicated. Abbreviated Canine Chronic Enteropathy Clinical Activity Index (CCECAI) scores were assessed at four different time points in **b** DR (*n* = 20) and **c** NDR (*n* = 9) animals. **d** Ternary plot of phylum-level OTUs from top 5 most abundant phyla among healthy (right), DR (left), and NDR (top) animals. Only OTUs with a maximum relative abundance > 0.001 and existing in at least 10% samples are shown. Bubble size represents the log2-transformed mean abundance of each OTU. Axes show reads accounted for by each OTU in each group of animals (DR, NDR, and healthy), as a percentage of total (sum) reads observed for a given OTU across all three groups. Arrows indicate the corresponding axis directions for each point. Relative abundance of **e** *E. coli* and **g** *C. perfringens* in animals with active disease (day 0) and healthy dogs. Spearman’s correlation between log10 abundance of **f** *E. coli* or **h** *C. perfringens* and CCECAI disease score. The shaded areas show the 95% confidence interval, as implemented in the geom_smooth function in the ggplot2 R package. **i** Differentially abundant OTUs between DR and NDR animals at day 0. *Y*-axis value represents the log2 fold change for DR versus NDR. *P < 0.05, **P < 0.01, ***P < 0.001, ****P < 0.0001 using the two-sided Wilcoxon signed-rank test. Spearman’s correlations in **f** and **h** are significant (*P* < 0.05) with correlation coefficients of 0.216 and 0.227, respectively

Twenty-nine dogs with CE were enrolled in a study to evaluate the Effect of Nutritional Therapy on Microbiome in Canine Enteropathy (the “ENTiCE” study). Animals with active disease were switched from their current diet to a commercially available therapeutic hydrolyzed protein diet (Fig. 1a). Impact of treatment on disease was monitored using the Canine Chronic Enteropathy Clinical Activity Index (CCECAI; hereafter referred to as “disease score”), which is positively correlated with poor clinical outcome [37]. After 2 weeks on therapeutic diet, 69% (20/29) of animals entered remission, marked by a reduction in the mean disease score from 4.1 (95% CI = 4.8–3.3) to 1.3 (95% CI = 1.8–0.7). These diet-responsive (DR) animals (dogs with FRE) were maintained on diet for the remainder of the study with no additional interventions (Fig. 1a). At the conclusion of the study (day 42), DR animals had a mean disease score of 0.9 (95% CI = 1.3–0.6), constituting a > 4-fold reduction in disease severity compared to day 0 (Fig. 1b). In contrast, 31% (9/29) of animals failed to show a significant reduction in disease score after 2 weeks on therapeutic diet (Fig. 1c). These non-diet-responsive (NDR) animals presented with more severe disease scores (mean score = 6.1; 95% CI = 7.4–4.7) than DR animals (*P* < 0.05 at day 0) and did not show a significant reduction 2 weeks after starting diet therapy (Fig. 1c). NDR animals were maintained on therapeutic diet for the remainder of the study, while receiving combination therapy that included antibiotics (beginning at day 14) and prednisone (beginning at day 28) (Fig. 1a and Additional file 1: Figure S1, see the “Methods” section), but showed only incremental improvement in disease scores (Fig. 1c). These data highlight a rapid and sustained clinical response to hydrolyzed diet in the majority of dogs with CE.

### Identification of microbial community profiles associated with treatment outcome

To determine whether treatment with hydrolyzed diet alone altered the microbial community in the gut, 16S rRNA gene profiling was carried out on fecal samples collected from DR (*n* = 20), NDR (*n* = 9), and healthy control animals at baseline (*n* = 24). Consistent with previous reports [38], we found that the species diversity of the canine fecal microbiome was not dramatically altered in dogs with CE compared to healthy controls (Additional file 1: Figure S2A-B) and that the communities in both groups were predominantly comprised of *Firmicutes*, *Bacteroidetes*, *Proteobacteria*, *Actinobacteria*, and *Fusobacteria* (Additional file 1: Figure S2C). However, compared to healthy dogs, animals with CE showed greater between-individual distance in microbial community structure by weighted UniFrac (Additional file 1: Figure S2D). Using a ternary plot visualization, we observed an enrichment of operational taxonomic units (OTUs) from *Proteobacteria* and a subset of OTUs from *Firmicutes* in animals with active disease at day 0 (Fig. 1d, green and tan points toward left side of triangle; Additional file 1: Figure S3). Interestingly, a subset of proteobacterial OTUs was highly enriched in DR animals compared to both NDR and healthy controls (Fig. 1d, tan points in lower left corner).

These differences prompted us to carry out a formal differential abundance analysis, identifying 84 OTUs that distinguish healthy animals from those with disease (Additional file 2: Table S1 and Additional file 1: Figure S3). For example, *Escherichia coli*, which is commonly associated with intestinal disease, was overrepresented in animals with CE (Fig. 1e), showing a significant, albeit weak, positive correlation with disease score (*R* = 0.22, *P* = 0.02) (Fig. 1f). OTUs from *Clostridium* sensu stricto 1 were also enriched in CE, including *Clostridium perfringens* (Fig. 1g), which was also positively correlated with disease scores (Fig. 1h) (*R* = 0.23, *P* = 0.016) and which has been implicated in large bowel diarrhea/colitis in dogs [39]. Taken together with previously published work [40, 41], these data demonstrate that dysbiosis during CE is marked by the presence of pathobionts, such as *E. coli* and *C. perfringens*. Next, we asked whether the microbiome in DR and NDR animals differed prior to starting treatment (day 0). Although no differences were observed between the two groups in community diversity, evenness, or distance from healthy controls (unweighted or weighted UniFrac), we identified 20 OTUs that were differentially abundant between DR and NDR animals, 12 of which were enriched in animals that ended up responding to diet treatment (Fig. 1i and Additional file 2: Table S2). Interestingly, *Proteobacteria* were found to be more abundant in DR animals (Fig. 1i). Collectively, these results reveal distinct microbial signatures during disease that are associated with different clinical outcomes following the dietary therapy.

### Therapeutic diet reduces dysbiosis associated with chronic enteropathy

**Fig. 2:**
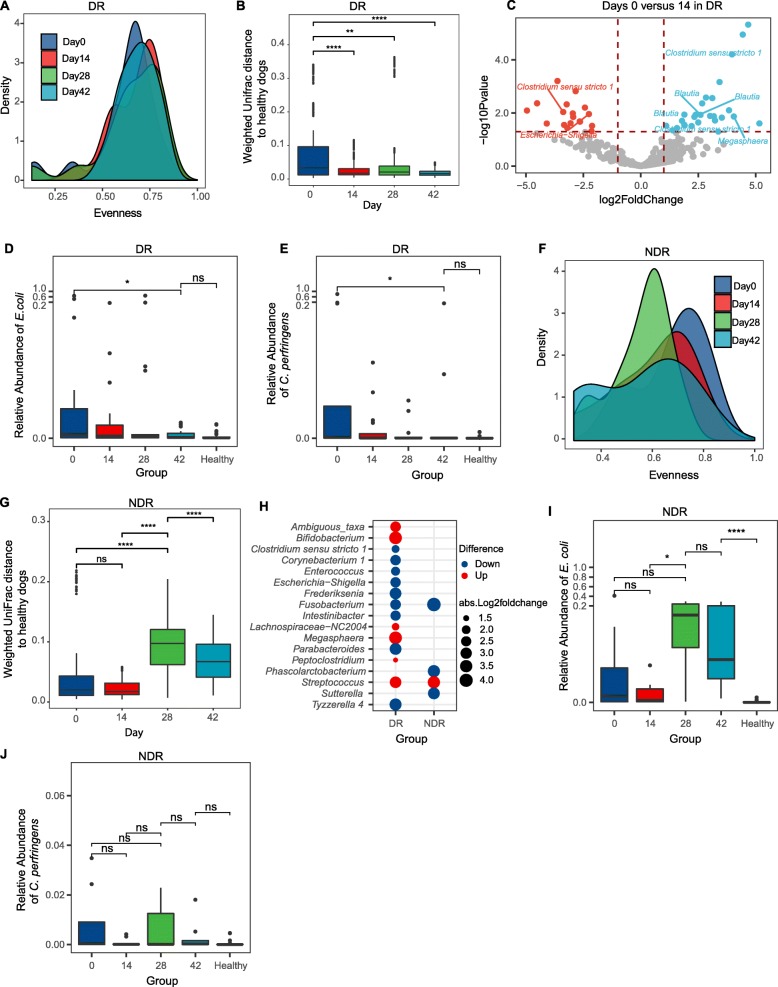
Therapeutic diet ameliorates dysbiosis associated with chronic enteropathy and induces changes in the microbiome associated with remission. **a** Density of Pielou’s evenness index for DR animals at different time points in the study. **b** Weighted UniFrac distance to healthy controls for DR animals. **c** Volcano plot showing differentially abundant OTUs enriched in either DR dogs with active disease (day 0, red points) or in remission after diet therapy (day 14, blue points). Selected taxa (e.g., *Escherichia-Shigella spp.*, *Clostridium spp.*, and *Blautia*; OTUs with mean abundance > 5 counts) are labeled. The relative abundance of **d** *E. coli* and **e** *C. perfringens* in DR animals throughout the study and compared to healthy controls. **f** Density of Pielou’s evenness index in NDR animals. **g** Weighted UniFrac distance to healthy dogs for NDR animals. Diet therapy began at day 0, metronidazole administration at day 14, and prednisone at day 28 (see the “Methods”). **h** Bubble plot showing differentially abundant genera (fold change > 2 and *P* < 0.05) between day 14 versus day 0 for DR (left) and NDR (right) animals. Bubble size indicates absolute log fold change between day 14 and day 0, and color reflects direction of change. **i**, **j** The relative abundance of *E. coli* and *C. perfringens* in NDR animals throughout the study and compared to healthy controls. The distribution comparison of density (**a**, **f**) was performed by comparing quantiles via the Harrell-Davis estimator (FDR < 0.01). ns, not significant. *P < 0.05, **P < 0.01, ***P < 0.001, ****P < 0.0001 using the two-sided Wilcoxon rank sum test for **b** and **g** or using the two-sided Wilcoxon signed-rank sum test for panels **d**, **e**, **i**, and **j**

To assess whether the diet-induced remission was accompanied by alterations in dysbiosis, we compared the microbial community structure before and after administration of therapeutic diet in DR animals. Although global phylogenetic distance and Shannon diversity were unchanged (Additional file 1: Figure S4A-B), a measurable increase in community evenness was observed following 14 days of diet therapy (Fig. 2a and Additional file 1: Figure S5), when focusing on the top 40 most abundant OTUs among the samples, which account for 83% of the total reads. Principal coordinate analysis (PCoA) based on unweighted (Additional file 1: Figure S4C) or weighted (Additional file 1: Figure S4D) UniFrac showed a measurable separation between dogs, even at day 0, before diet was administered. Despite this baseline difference between animals, community structure underwent a shift away from disease state by 14 and 42 days after diet therapy (Additional file 1: Figure S4C and D). This shift is significantly higher than the heterogeneity within healthy group, suggesting an influence from diet therapy (Additional file 1: Figure S4E). Comparing weighted (Fig. 2b) or unweighted (Additional file 1: Figure S4F) UniFrac distances between DR and healthy animals at each time point, we observed that diet-induced remission was accompanied by decreased distance relative to healthy controls, a trend that continued through day 42, when the similarity to day 0 was lowest and similarity to healthy dogs was highest (Fig. 2b).

Given that therapeutic diet is associated with a shift in community structure of the microbiome in DR animals, we reasoned that composition of the fecal microbiome would be rapidly altered after dietary intervention. Administration of therapeutic diet was broadly characterized by an increase of *Firmicutes* and a decrease of *Proteobacteria* (Additional file 1: Figure S4G and H). Fourteen days after beginning diet therapy, 14 genera were differentially abundant compared to pretreatment (day 0) in DR animals (Additional file 2: Table S3). Potentially pathogenic genera associated with IBD were underrepresented after diet treatment in DR animals. For example, *Escherichia-Shigella*, *Clostridium* sensu stricto 1, *Enterococcus*, and *Fusobacterium* had a higher relative abundance at day 0 and were significantly reduced after 14 days on therapeutic diet. When evaluated at the species level, 45 OTUs were significantly differentially abundant between samples collected at day 0 compared to day 14 (Fig. 2c, Additional file 2: Table S4). *E. coli* was enriched in animals at day 0 in this study (Fig. 2d), and relative abundance of this taxon declined dramatically after 2 weeks on therapeutic diet, eventually reaching levels nearly undetectable by day 42 that were also indistinguishable from levels observed in healthy dogs (Fig. 2d). *C. perfringens* also showed a significantly lower prevalence in the samples at day 14 and in healthy dogs, compared to day 0 samples (Fig. 2e). In contrast, OTUs from *Blautia* were increased after diet therapy, consistent with reports that members of this genus are beneficial commensals [42] (Additional file 2: Table S4). Taken together, these results point to ameliorated dysbiosis with a reduction of pathobionts and increase of beneficial commensal taxa as a hallmark of the diet therapy.

### Remission-specific changes in the microbiome following diet therapy

We hypothesized that the changes observed following diet therapy in DR animals would be associated with remission, rather than merely a response to diet that is independent of clinical outcome. To test this hypothesis, we compared the impact of therapeutic diet on DR dogs with changes observed between days 0 and 14 in NDR animals that failed to enter remission after diet therapy, and which required additional therapies after day 14. Whereas 14 days of diet therapy in DR animals was associated with increased community evenness (Fig. 2a) and a decreased distance from healthy dogs (Fig. 2b), the same treatment in NDR dogs did not significantly affect the microbial community evenness (Fig. 2f, red) or weighted (or unweighted) UniFrac distance to healthy dogs (Fig. 2g and Additional file 1: Figure S4I). Just as we observed in DR animals (Additional file 1: Figure S4G), diet also altered the gut microbiota compositions in NDR animals (Additional file 1: Figure S4J). However, this shift was distinct from that observed in DR animals (Fig. 2h). Differential abundance analysis comparing NDR animals at day 0 versus day 14, during which time they received only therapeutic diet, identified 23 OTUs (Additional file 2: Table S5). Diet therapy in NDR animals was associated with a decrease in the relative abundance of some taxa, such as *Fusobacterium* and *Phascolarctobacterium* taxa, that were either unchanged or more modestly altered by diet therapy in DR animals. Conversely, *Escherichia-Shigella*, *Enterococcus*, and some of *Clostridium* sensu stricto 1 were not reduced in animals that failed diet therapy (Fig. 2h). Similarly, *E. coli* and *C. perfringens* were not significantly reduced in NDR animals after diet therapy (Fig. 2i, j). After 14 days on therapeutic diet, NDR dogs were maintained on diet, but were also administered metronidazole, an antibiotic that largely targets anaerobes. As expected, antibiotic treatment exacerbated dysbiosis, resulting in a precipitous decline in community evenness (Fig. 2f, green), increased distance from healthy controls (Fig. 2g, green), and increased relative abundance of *E. coli* and *C. perfringens* (Fig. 2i, j, green). Interestingly, steroid treatment (initiated on day 28 for NDR animals) mildly ameliorated the dysbiosis, resulting in decreased distance to healthy dogs (Fig. 2g, light blue).

### Diet-induced remission is associated with metabolic reprogramming and increased levels of secondary bile acids

**Fig. 3:**
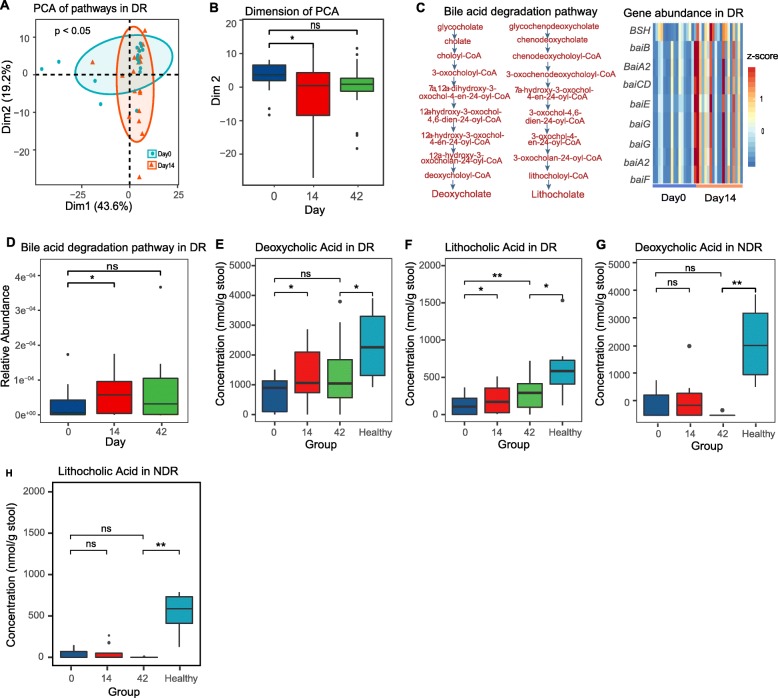
Diet-induced remission is associated with metabolic reprogramming and increased levels of secondary bile acids. **a** PCA analysis of UniPathway pathways based on the results of HUMAnN2 analysis for DR animals. **b** The first principal component (dim 2) from **a**, for all time points. **c** Heatmap showing the relative abundances of genes in secondary bile acid biosynthesis pathway after diet in DR animals. **d** Relative abundance of the pathway for secondary bile acid biosynthesis, predicted based on metagenomic data for DR animals. **e–h** Levels of deoxycholic (**e**) and lithocholic (**f**) acid measured in the stool of DR animals and NDR animals (**g**, **h**). ns, not significant. *P < 0.05, **P < 0.01 using Hotelling’s T-squared test for **a** or the two-sided Wilcoxon signed-rank test for other panels

To determine if the changes in microbial community structure would translate to altered microbial metabolism, stool samples collected day 0, 14, and 42 after starting diet therapy were subjected to metagenomic sequencing. Principal component analysis of metabolic pathway abundance data showed a separation (*P* < 0.05) between DR animals before and after diet-induced remission (days 0 vs. 14) (Fig. 3a, b). Differential abundance analysis identified 35 global pathways and 120 pathways at the species level that were significantly altered in relative abundance as a result of diet treatment in DR animals (Additional file 2: Table S6), including several involved in carbohydrate metabolism and secondary bile acid synthesis, prompting us to quantify bile acids in stool. Using targeted metabolomics, we measured the levels of 15 bile acids in stool of healthy dogs, compared with days 0, 14, and 42 (Additional file 1: Figure S6). Consistent with increased relative abundance of bile salt hydrolase (BSH) and bile acid-inducible operon (bai) genes (Fig. 3c and Additional file 1: Figure S7) involved in secondary bile acid synthesis (Fig. 3d), the secondary bile acids deoxycholic acid (Fig. 3e) and lithocholic acid (Fig. 3f) were reduced in animals with active disease (day 0), compared to healthy dogs, and were significantly increased after the diet treatment in DR animals at day 14 and 42 (Fig. 3e, f, and Additional file 2: Table S7). Notably, secondary bile acids were not elevated by diet treatment in NDR animals (Fig. 3g, h), suggesting that this metabolic shift is associated with remission.

### Lithocholic and deoxycholic acid inhibit the in vitro growth of *E. coli* and *C. perfringens*

**Fig. 4:**
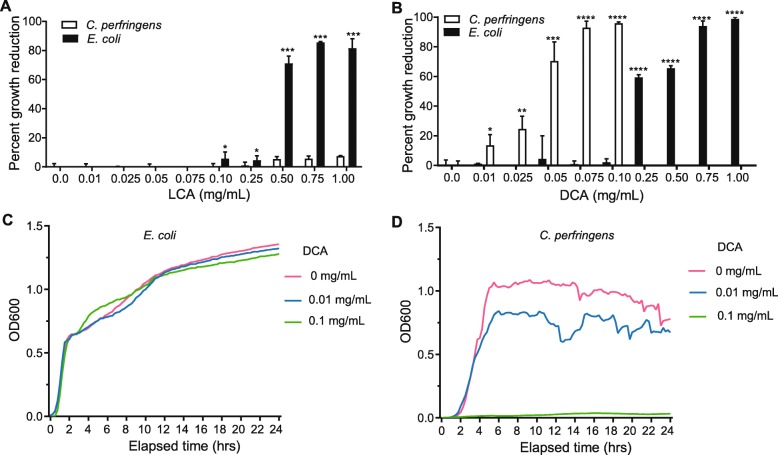
Secondary bile acids inhibit the expansion of potential pathogens in vitro. **a**, **b** In vitro growth of canine clinical isolates of *E. coli* or *C. perfringens* in the presence of varying concentrations of lithocholic acid or deoxycholic acid (mean ± sd shown). Each point in the graphs represents one replicate in the assay. As *E. coli* had a full growth in lithocholic acid < 0.075 mg/mL and *C. perfringens* had no growth in deoxycholic acid > 0.1 mg/mL, these assays were not determined below and above these concentrations, respectively. **c**, **d** Growth curves of deoxycholic acid on *E. coli* and *C. perfringens*. The in vitro inhibition tests for **a** and **b** were repeated in three independent experiments, and data shown are from a representative experiment. **c** and **d** show the mean values of three technical replicates. *P < 0.05, **P < 0.01, ***P < 0.001, ****P < 0.0001 using two-sided *t* test

Given that bile acids have emerged as potential regulators of the gut microbiome, we hypothesized that the secondary bile acids observed in our study could impact microbiome structure [43]. Correlation analysis of our metabolomics and microbiome data reveal that the primary bile acid, cholic acid, was negatively correlated with 10 OTUs (Additional file 2: Table S8), consistent with the reported ability of this bile acid to broadly and negatively regulate bacterial growth [43]. We also observed that the increase in secondary bile acids following diet treatment correlated with reduced relative abundance of certain bacteria (e.g., OTUs from *Escherichia-Shigella*, *Clostridium*, and *Fusobacterium*) (Additional file 2: Table S8). To directly test whether secondary bile acids could have a negative impact on potential pathogenic bacteria from the canine gut, lithocholic and deoxycholic acid were assessed for their ability to inhibit the in vitro growth of *E. coli* (*n* = 1) or *C. perfringens* (*n* = 3) isolates derived from dogs with active CE in our ENTiCE study. These species were selected since either they or their parent genera were associated with disease in our animal model. Deoxycholic acid blocked the growth of both species at a concentration comparable to what we detected in the fecal samples (in DR animals at day 42, mean = 0.43; 95% CI 0. 22–0.63 mg/g) (Fig. 4b and Additional file 1: Figure S6), while lithocholic acid (in DR animals at day 42, mean = 0.09; 95% CI 0.05–0.13 mg/g) modestly blocked the growth of *E. coli* but not *C. perfringens* (Fig. 4a). In contrast, *C. perfringens* is much more sensitive to deoxycholic acid than *E. coli* (Fig. 4c, d). Collectively, these results show that the inhibitory activity of these secondary bile acids varies for different bacteria and suggest that elevated lithocholic and deoxycholic acids observed following the dietary therapy may contribute to the control of harmful bacteria.

### *C. hiranonis* is a diet-responsive species with the ability to produce secondary bile acids

**Fig. 5:**
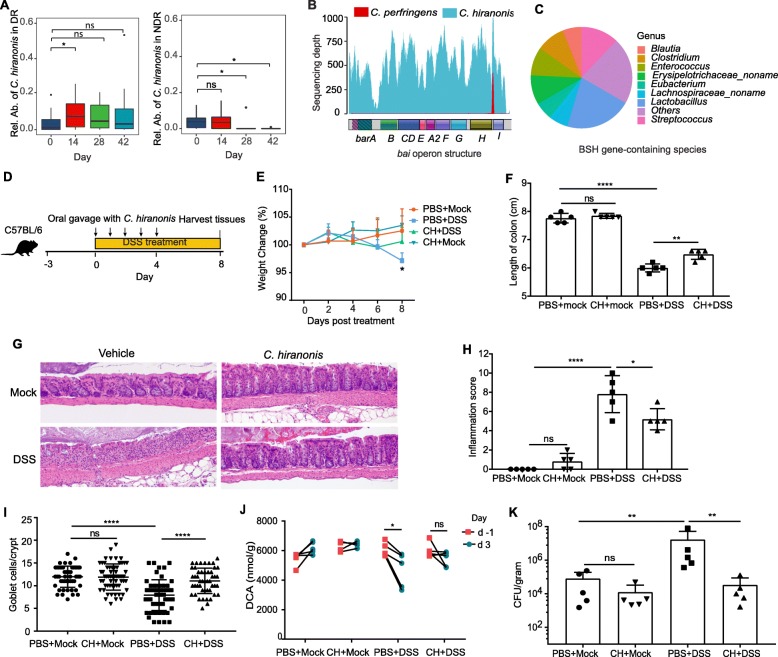
*C. hiranonis* is a diet-responsive species with the ability to ameliorate intestinal disease. **a** Relative abundance of the OTU corresponding to *C. hiranonis* (HQ776819.1.1426) in 16S rRNA sequencing data for DR and NDR animals. **b** Coverage of the bile acid operon (*bai*) from the *C. hiranonis* reference (ASM15605v1) with whole genome sequencing reads produced *C. hiranonis* (teal) and *C. perfringens* (red) canine clinical isolates. **c** Bile salt hydrolase (BSH) gene-containing species in DR animals. Pie chart shows the proportion of (BSH) gene-containing species from each genus. **d** Schematic showing experimental design for mouse experiments. **e** Weight changes across the experiment (*n* = 5). **f** Length of colon at day 8. **g** Representative H&E staining sections of distal colon tissues at day 8 (× 20 objective) (*n* = 5 mice). **h** Inflammation scores estimated from distal colon tissue with H&E staining (*n* = 5). **i** Goblet cell numbers in crypts of distal colon, identified with PAS staining. Ten crypts in each section were randomly selected and evaluated only if aligned along the longitudinal axis such that the lumen of the crypt could be seen along its length. **j** DCA concentration in mouse stools before (day -1) and after (day 3) DSS treatment (*n* = 4 or 5 mice). **k** Colony-forming units (CFUs) of *E. coli* in colon content of mice (*n* = 5). *E. coli* was colonized into mice 3 days before DSS treatment (day -3), and the colon lumen content was harvested at day 8. Experiments (**d**–**k**) were repeated at least 2 times with similar results. Data shown are from a representative experiment (mean ± sd shown). ns, not significant. *P < 0.05, **P < 0.01, ****P < 0.0001 using the two-sided Wilcoxon signed rank sum test for **a** and **k**, and the two-sided *t* test for **e**, **f**, **h**, **i**, and **j** (paired test for **j**)

Next, we sought to identify potential sources of lithocholic and deoxycholic acids after diet treatment. Conversion of primary bile acids to these secondary bile acids requires the 7-dehydroxylation activity conferred by the bile acid-inducible (*bai*) operon—an activity unique to a limited number of anaerobes representing a small fraction of the microbiome, including some Clostridial and Eubacterial species [44]. Given our finding that certain clostridial (or pepto-clostridial) OTUs, as well as levels of lithocholic and deoxycholic acids, increase after diet-induced remission, we set out to identify potential bile acid producers in DR animals. Taxonomic assignment of metagenomic reads identified six *Clostridium* species (*C. perfringens*, *C. hiranonis*, *C. nexile*, *C. colicanis*, *C. glycolicum*, and *C. ramosum*) and two *Eubacterium* species (*Eubacterium biforme* and *E. dolichum*) present in these samples at a relative abundance ≥ 0.01% in at least 10% samples. Of these species, *C. hiranonis* was among the most abundant (Additional file 1: Figure S8) and is the only one reported to have the *bai* operon [45]. Moreover, our metagenomic data (Additional file 1: Figure S9) showed that the relative abundance of *C. hiranonis* was significantly increased after diet treatment in DR animals (Fig. 5a, left) but not in NDR animals that failed diet therapy (Fig. 5a, right). Since the *Clostridium* genus exhibits a high level of genetic divergence, even at the species level, we set out to confirm that canine *C. hiranonis* possesses the *bai* operon. Anaerobic culture of rectal swabs collected during the study, followed by random isolate picking and Sanger sequencing of full-length 16S rRNA gene, was used to assemble a canine culture collection from 7 dogs with CE before and/or after treatment. In total, 49 *Clostridium* isolates belonging to 5 species were identified (*C. baratii*, *C. perfringens*, *C. sartagoforme*, *C. hiranonis*, and *C. lactatifermentans*). Eighty-two percent (31/39) of the clostridial isolates from animals with active disease were *C. perfringens*, consistent with the reported involvement of this organism in canine [39] and human [46] gastrointestinal disease. Two *C. hiranonis* isolates were obtained from independent DR animals in remission at day 42. We selected these *C. hiranonis* isolates and three *C. perfringens* isolates for whole genome sequencing. Reads were aligned to the reference *C. hiranonis* genome, revealing an intact *bai* operon in canine *C. hiranonis*, but not *C. perfringens* (Fig. 5b). In addition, the reduced abundance of *C. hiranonis* (Fig. 5a) after antibiotic treatment in NDR group was associated with a decrease in the level of secondary bile acids (Fig. 3g, h) in stool of these animals. As an important component in bile acid degradation, bile salt hydrolases (BSHs) [47] were examined. Thirty-one BSH-containing species were identified in our metagenomic data, the majority of which belong to the genera *Lactobacillus*, *Streptococcus*, and *Eubacterium* (Fig. 5c). Several of these species are significantly positively correlated with the lithocholic acid or deoxycholic acid (Additional file 2: Table S9 and Additional file 1: Figure S7B), highlighting that BSH-containing microbes may contribute to increased levels of these bile acids. Collectively, these data point to *C. hiranonis*, a species originally isolated from human stool [45], as the most likely secondary bile acid producer associated with the diet-induced remission in dogs.

The association between *C. hiranonis*, secondary bile acids, and remission in CE prompted us to test whether *C. hiranonis* could ameliorate disease in a mouse model of colitis. Inflammation and dysbiosis were triggered by the administration of dextran sodium sulfate (DSS) in drinking water [48], and animals were either orally administered PBS or *C. hiranonis* (Fig. 5d). As expected, DSS treatment resulted in significantly reduced weight (Fig. 5e), colon length (Fig. 5f), gut damage marked by infiltration of mononuclear cells and neutrophils into the lamina propria (Fig. 5g, h; Additional file 1: Figure S10), and hyperplastic crypts with increased numbers of mitotic figures and reduced goblet cell numbers (Fig. 5g, i). In addition, DSS treatment resulted in bile acid dysregulation marked by reduced levels of deoxycholic acid in the stool (Fig. 5j). In contrast, DSS-treated mice that received *C. hiranonis* showed a marked reduction of colonic shortening (Fig. 5f), a less severe inflammation (Fig. 5g, h) that was restricted to the lamina propria and which involved fewer neutrophils, a maintenance of goblet cell numbers (Fig. 5g, i), and a preservation of deoxycholic acid levels (Fig. 5j). In addition, when mice were precolonized with a food-safe strain of *E. coli* (Nissle 1917) at day -3, DSS treatment resulted in significant bacterial expansion (dysbiosis) in mice by day 8 (Fig. 5k). This expansion was markedly reduced in mice that received *C. hiranonis*. Taken together with the results from the canine model, these data suggest that *C. hiranonis* is a beneficial microbe implicated in remission of intestinal disease.

### *C. scindens* is associated with diet-induced remission in pediatric Crohn’s disease

**Fig. 6:**
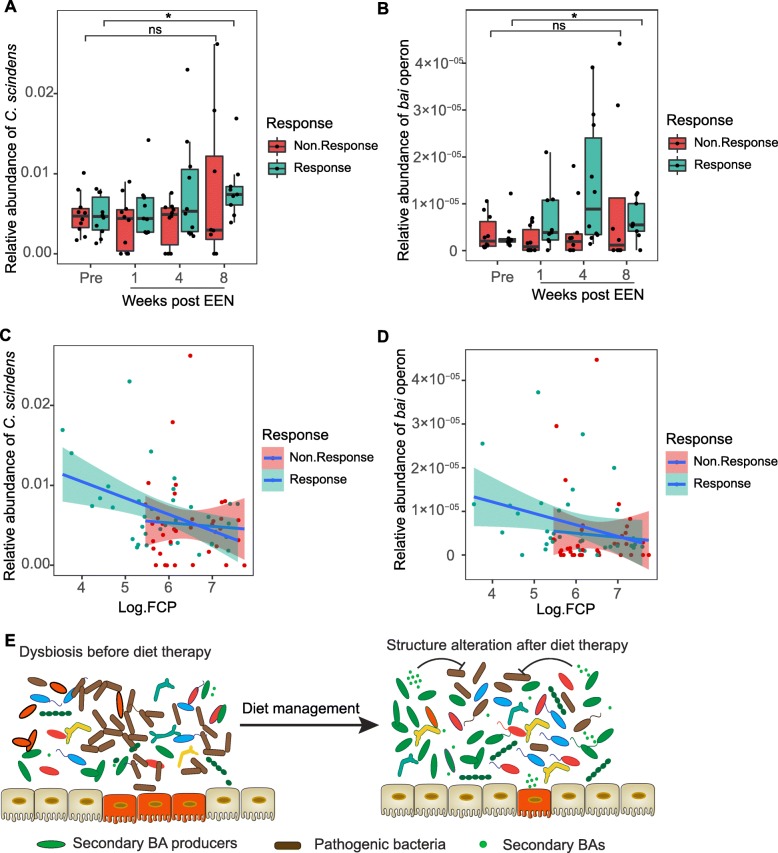
The bile acid producer, *C. scindens*, is associated with diet-induced remission in human pediatric Crohn’s disease. Analysis of public data [23] from human pediatric Crohn’s disease patients treated with exclusive enteral nutrition (EEN). Relative abundance of reads (mapping ratio) aligning to **a** *C. scindens* reference or **b** *bai* operon from 20 patient’s pretreatment and 1, 4, and 8 weeks following administration of EEN. Patients that responded to treatment and entered remission (*n* = 10, green) and those that failed therapy (*n* = 10, red) are shown. **c**, **d** Spearman’s correlations between log10-transformed fecal calprotectin levels (FCP) and relative abundance of *C. scindens* (*R* = − 0.351 for “Responsive,” *P* = 0.033; *R* = − 0.027 for “Non.Responsive,” *P* = 0.877) or *bai* operon (*R* = − 0.394 for “Responsive,” *P* = 0.016; *R* = 0.049 for “Non.Responsive,” *P* = 0.777). The shaded areas show the 95% confidence interval. **e** Schematic showing proposed model for microbiome alterations. ns, not significant. *P < 0.05, **P < 0.01, ***P < 0.001 using the two-sided Wilcoxon rank sum test for relative abundance comparisons

Given that high remission rates are observed in both dogs and humans following diet therapy, we hypothesized that a similar induction of *bai* operon-containing clostridia may occur in pediatric Crohn’s disease patients being treated with exclusive enteral nutrition (EEN). To test this, we analyzed publicly available data from the Pediatric Longitudinal Study of Elemental Diet and Stool Microbiome Composition (“PLEASE”), a study that examined approximately 20 patients before and after treatment with EEN [23], in which half responded to treatment while the other half failed EEN therapy. Classifications of bacterial taxa present in each sample using metagenomic analysis methods [49] revealed the presence of *C. scindens*, which like *C. hiranonis* is also recognized for having high 7-dehydroxylation activity [45, 50]. The relative abundance of *C. scindens* was estimated using the proportion of total reads that map to the reference genome, revealing significant increases in this species and the *bai* operon from pretreatment to 8 weeks post-EEN (Fig. 6a, b, respectively). Remarkably, this increase was observed in patients that entered remission following EEN (responsive, *n* = 10) but not those that failed therapy (non-responsive, *n* = 10) (Fig. 6a, b). In addition, the correlation analysis between the relative abundance of *C. scindens* and fecal calprotectin (FCP), a biomarker of disease activity for IBD [23], indicated a significantly negative correlation (Fig. 6c) in diet-responsive patients (*R* = − 0.3515, *P* = 0.03287), but not in non-responders (*R* = − 0.0267, *P* = 0.877). Similarly, a significant negative correlation between *bai* operon and FCP was observed in diet-responsive (*R* = − 0.3944, *P* = 0.0157), but not non-responsive patients (*R* = 0.0490, *P* = 0.7766) (Fig. 6d). These results show that bile acid-producing clostridia are associated with diet-induced remission in both animals and humans alike (Fig. 6e).

## Discussion

Using an animal model of food-responsive chronic inflammatory enteropathy, we showed that secondary bile acids and bile acid-producing *Clostridium* species (*C. hiranonis* and *C. scindens*) are key features associated with diet-induced remission in humans and dogs. Previous studies reported dysregulated bile acid metabolism in IBD patients [51] and dogs with CE [52], and showed that *C. hiranonis* and *E. coli* were biomarkers of disease status in dogs [53]. Our results extend these observations by identifying *C. hiranonis* as a remission-associated taxon that may serve as a source of secondary bile acids. Furthermore, we observed that *C. hiranonis* preserved deoxycholic acid levels and ameliorated disease in DSS-treated mice, further highlighting a link between bile acid-producing clostridia and remission of gastrointestinal diseases. Our in vitro inhibition experiments with lithocholic and deoxycholic acids on the *C. perfringens* and *E. coli* isolates, together with previous findings of in vitro antibacterial activities of lithocholic acid on *E. coli* [54, 55], suggest that the increased *C. hiranonis* abundance could potentially contribute to the control of pathobionts in the gut. Indeed, as dogs entered remission following dietary therapy, we observed a remarkable decrease of *C. perfringens* and *E. coli*, coincident with increased levels of lithocholic and deoxycholic acid. In addition to being antimicrobial, physiological levels of these bile acids have been shown to modulate intestinal inflammation, in part due to their ability to signal through the farnesoid X receptor and other bile acid receptors to inhibit pro-inflammatory signaling [55–57]. Our data point to specific bile acids as being strongly associated with diet-induced remission, and warrant additional mechanistic studies to determine whether and how bile acid-producing clostridia modulate chronic inflammatory enteropathies in vivo.

Our findings complement recent studies showing that bile acid-producing clostridia mediate protection against *C. difficile* infection [58]. *C. difficile* infections frequently arise after antibiotic treatment, a phenomenon attributed to the effect of antibiotics on secondary bile acid levels [59]. Interestingly, we also observed that antibiotics antagonized the diet-induced shifts in microbiome composition and function, promoting a more dysbiotic state coincident with dramatically reduced levels of lithocholic and deoxycholic acid and elevated levels of *E. coli* (Figs. 2 and 3). Taken together with the antibacterial activities of secondary bile acids (Fig. 4), these data imply a possible general model for microbe-microbe interactions in the gut in which bile acid-producing clostridia may restrict the growth of a range of bile acid-sensitive pathobionts to limit disease, and highlight that these processes are exquisitely sensitive to antimicrobials. This model will need to be elaborated by in vitro and in vivo assays, since our data show that the extent to which bile-acid producers can restrict pathobiont growth will likely be dependent on the specific bile acid as well as the target microbe (Fig. 4). At physiologic levels, deoxycholic acid was more effective than lithocholic acid at restricting the growth of *E. coli* and *C. perfringens*. Yet, *E. coli* was notably more resistant, requiring concentrations of deoxycholic acid 10 times higher than what was needed to restrict *C. perfringens*. Several mechanisms mediating bile acid resistance have been described for *E. coli*, including the outer membrane acting as a barrier to the influx of bile salts [60], and activation of efflux pumps [61] and the SOS response [62]. However, little is known about the response of Clostridial species to bile acid stress, but differences in cell wall composition and physicochemical properties, such as hydrophobicity, between gram-negative and gram-positive bacteria may play a role. In addition, the observation of strain-specific variability in resistance to bile acids has been described for *C. difficile* [63], suggesting genetic factors may be involved. The poor solubility of many bile acids, together with evidence that supra-physiologic levels of bile acids are pathologic [64, 65], makes it challenging to directly manipulate the microbiome by administration of these compounds. However, our results showing that gavaging mice with *C. hiranonis* stabilize levels of deoxycholic acid during colitis suggest a possible indirect and safe way for manipulating intestinal bile acids. Whether or how *C. hiranonis* or secondary bile acids play a role in restricting *E. coli*, *C. perfringens*, or other pathobionts in vivo remains to be determined, but elucidating these mechanisms might have important health implications beyond veterinary medicine. Interestingly, when we examined data from a cohort of pediatric Crohn’s disease patients before and after diet therapy, we found that *C. scindens*, a well-known secondary bile acid producer, was associated with diet-induced remission (Fig. 6). This is particularly relevant to our findings given a recent report that sustained remission following EEN was characterized by low levels of proteobacteria, while patients that relapse showed a marked increase in *Proteobacteria* [66]. Differences in the species of secondary bile acid producers observed between dogs and humans may be shaped by host-microbe interactions, but it is also possible that other *bai* operon- or BSH gene-containing species make important contributions to the secondary bile acid production and pathobiont restriction in vivo.

Rates of remission achieved with diet therapy in this and prior studies of canine CE [37] mirror remission rates achieved with EEN for treating pediatric Crohn’s disease [17]. In both cases, however, a substantial portion of patients fail to respond to therapy. Understanding why some patients fail and what might be done to improve remission rates during diet therapy is critical in improving clinical care for inflammatory bowel diseases. Treatment outcome was not associated with differences in baseline levels of *C. hiranonis*, as both DR and NDR animals in our study had comparable levels of *C. hiranonis* prior to treatment, suggesting that microbe-microbe interactions, differing mucosal immune responses, or other factors may be necessary to support expansion of *C. hiranonis* during diet therapy. Compared to diet-responsive animals, those that failed treatment presented with higher overall disease scores. At first glance, this would seem to contradict evidence from human studies, where EEN achieved higher remission rates in children with a more severe disease (based on the Pediatric Crohn’s Disease Activity Index, or PCDAI) and resulted in more mucosal healing (reduced fecal calprotectin levels) [17]. However, closer inspection of disease scores in our study (Additional file 1: Figure S11A) showed that NDR animals presented more frequently with vomiting and inappetence than their DR counterparts, indicating that these symptoms may limit the effectiveness of diet as therapy and raising the possibility that antiemetics and/or appetite stimulants could improve the efficacy of diet for some patients. The microbial and metabolic differences we observed between DR and NDR animals were still evident when only score-matched animals were considered in the analysis (Additional file 1: Figure S11), suggesting that the microbiome phenotype we have described is associated with treatment outcome rather than disease severity. Taken together, these data suggest that both clinical and microbial biomarkers could be used to predict patients that might be refractory to diet treatment.

The precise role that diet and dietary components play in remission has yet to be defined. One interpretation of our data is that therapeutic diet modifies the composition and/or function of the microbiome, which subsequently impacts pathobionts (e.g., *E. coli* and *C. perfringens*), host immunity, or both, thereby reducing pathology. Alternatively, diet may induce remission through mechanisms that are independent of the microbiome, in which case alterations to the microbiome and restoration of normal bile acid production would be consequences rather than causes of remission. Finally, diet may impact disease through both microbiome-dependent and microbiome-independent mechanisms. Nevertheless, given that certain bacteria and bile acids have been implicated in IBD pathogenesis and host inflammation [55], it seems unlikely that the reduced pathobionts and increased secondary bile acids have no contribution to remission in our model. Interestingly, a previous study showed that the composition of the gut microbiome was not affected when healthy dogs were fed a hydrolyzed diet [67]. Our data show that diet does, in fact, impact the microbiome of animals with disease, suggesting that therapeutic diets can have different effects depending on the basal state of the gut microbiome. Hydrolyzed protein diets, used in this study, have been shown to be effective in the management of canine CE [68, 69] and have previously been shown to be more effective for long-term management when compared to a highly digestible diet formulated with non-hydrolyzed protein sources [68, 69], but other studies have shown efficacy with non-hydrolyzed commercial prescription diets made from limited ingredients that include a novel protein source. Thus, one important open question is how therapeutic diets such as EEN or different prescription pet foods modulate disease and the microbiome and whether there are general principles that could be used to guide the development of better dietary interventions. Leveraging spontaneous animal models, such as the dog where formulated diets have long been a standard of care, will help dissect the therapeutic roles of different diets, thus providing valuable insight into human intestinal disease.

## Conclusions

In summary, these data show that remission induced by a therapeutic hydrolyzed protein diet is linked to improved microbiota structure in canine chronic inflammatory enteropathy, marked by decreased relative abundance of pathobionts and increased abundance of a secondary bile acid producer (*C. hiranonis*). Levels of lithocholic and deoxycholic acid in stool rose rapidly after the diet therapy and inhibited the in vitro growth of disease-associated taxa. Mining public data from diet therapy in human pediatric Crohn’s disease showed similar results, supporting a model by which dietary interventions lead to remission. These results warrant further investigations in other animal models and human studies and constitute an important first step in establishing a framework for evaluating the efficacy of dietary interventions, which could help guide the rational design of more effective therapeutic diets.

## Methods

### Diagnosis and treatment of canine chronic enteropathy

Client-owned animals presenting with clinical signs of canine chronic enteropathy (CCE) were screened at the Ryan Veterinary Hospital of the University of Pennsylvania for enrollment in a study to determine the Effect of Nutritional Therapy on Microbiome in Canine Enteropathy (“ENTiCE”). Dogs were screened if they had any one of the following clinical signs for ≥ 3 weeks’ duration: vomiting, diarrhea, or weight loss despite adequate caloric intake. Dogs were excluded from screening if they had been treated with a hydrolyzed protein diet, antibiotics, corticosteroids, or probiotics within the previous 2 weeks. At the time of screening, the following were performed on each animal: complete physical examination, routine fecal screening (including zinc sulfate flotation for parasite identification, gram stain and culture for *Salmonella* spp. and *Campylobacter* spp.), complete blood count, serum biochemical profile, serum measurement of canine trypsin-like immunoreactivity, cobalamin and folate, urinalysis, abdominal ultrasound examination, and disease severity scoring using the Canine Chronic Enteropathy Clinical Activity Index (CCECAI) [37]. If these initial screening tests failed to identify a cause for the clinical signs, upper and/or lower gastrointestinal endoscopy with mucosal biopsies was performed. Biopsies were fixed in formalin and embedded in paraffin, sections were stained with hematoxylin and eosin, and slides were examined by a board-certified veterinary pathologist. Dogs were enrolled only if histopathology revealed intestinal inflammation with no identifiable underlying cause (such as infectious agents). Dogs were excluded if another histopathologic diagnosis was identified.

The ENTiCE study included three 14-day treatment tiers (Fig. 1a and Additional file 1: Figure S1), and dogs were evaluated for a therapeutic response at the conclusion of each tier using CCECAI. Remission was determined using an abbreviated CCECAI that included scores to the first five indices (attitude/activity, appetite, vomiting, stool consistency, and stool frequency), and was defined as an abbreviated CCECAI score ≤ 2, with no score > 1 for any of the five indices. Animals were first administered a therapeutic hydrolyzed protein diet (Royal Canin Veterinary diet Hypoallergenic Adult dry dog). Dogs that entered remission following this treatment were designated as diet-response (DR) and were maintained on therapeutic diet for the reminder of the trial. Animals that did not respond to therapeutic diet (NDR) subsequently began a 2-week course of metronidazole (10 mg/kg PO q 12 h) while being maintained on the therapeutic diet. Dogs that entered remission following antibiotic treatment were maintained on the combination of antibiotics and therapeutic diet for the reminder of the trial. Animals that still failed to show a favorable response remained on diet and metronidazole, but received prednisone (1 mg/kg PO q 12 h) (tier 3) for the final 14 days of the trial. Dogs that presented with hypoalbuminemia (protein-losing enteropathy) at the initial screening were presumed to have more severe disease and poorer prognoses and thus were immediately administered all three interventions, and were not included in our analyses. All dogs in which serum cobalamin was low at screening were supplemented with cyanocobalamin (50 mcg/kg SQ q 7 days) for the duration of the study. At the conclusion of the study, all animals returned to the clinic for the primary endpoint, which included a full re-evaluation of dogs including complete physical examination, complete blood count, serum chemistry, serum measurement of cobalamin and folate (if low at screening visit), urinalysis, CCECAI scoring, and final fecal collections.

### 16S rRNA gene sequencing and data analysis

Genomic DNA was extracted from stool using the PowerSoil DNA Isolation Kit (MO BIO Laboratories, Carlsbad, CA) following the manufacturer’s recommendations. A mock community pool containing purified genomic DNA from 12 known bacterial isolates was amplified and sequenced as a quality control. Additional controls included extraction of blank-processed samples (in which the DNA extraction process was followed without addition of input material), and water only, to determine background microbial signal. A dual-index amplicon sequencing method was employed for PCR amplification of the V4 region of the 16S *rRNA* gene [70]. Pico-green-based amplicons were sequenced on a MiSeq platform (Illumina, San Diego, CA) using 250 bp paired-end chemistry. Reads were filtered to remove sequences with average Phred quality score ≤ 20 using Quantitative Insights into Microbial Ecology (QIIME) [71] with filtering options (-q 20 -p 0.75 -r 3). Homopolymers > 10 bp in length and sequences < 250 bp and > 256 bp were removed using Mothur [72]. Chimeric sequences were identified and removed by usearch61 [73] against the representative 16S sequences of SILVA128 (97_otus_16S.fasta) [74, 75]. Quality-controlled sequences were then clustered against the SILVA128 database (SILVA_128_QIIME_release) using the open-reference OTU picking as implemented in QIIME with default parameters. Analysis of OTU tables was carried out using the R statistical environment [76], the bioconductor suite of software [77], and the Phyloseq2 package [78]. Singletons and OTUs with ambiguous annotations were removed from the OTU table. The OTU table was rarefied to 10,600 sequences per sample. Alpha diversity (Shannon’s diversity index and Faith’s phylogenetic diversity) and beta diversity (weighted and unweighted UniFrac) were calculated using Phyloseq2. Pielou’s evenness index was calculated as previously described [79].

For differential abundance analysis and association analysis, filtering on un-rarefied OTU table of samples with > 10,000 reads was carried out to remove taxa with a max abundance < 0.1% across all samples and present in < 10% of all samples. DESeq2 [80] implemented in Phyloseq2 (test = “Wald,” fitType = “parametric”) was used for differential abundance analysis on different taxonomy levels (fold change > 2 and *P* value < 0.05) using un-rarefied reads. To estimate the effect from outliers, separate fold changes of relative abundances (1 read was added to avoid taking log of zero) were also calculated by excluding the 10% outliers (5th–95th) in each group. The Spearman correlation was computed between the abundance of each microbial composition log-transformed) and the values of different factors (i.e., CCECAI for each dog, time points, concentration of each metabolite). To avoid taking log of the zero value, we added 1 read to the abundance for each composition before calculating the Spearman correlation. All *P* values in the above analysis were adjusted by the FDR (Benjamini-Hochberg) method for multiple comparisons except where noted. In order to get a taxonomic assignment at species level for the OTUs from *Clostridium* sensu stricto 1, the corresponding representative sequences in SILVA database were used to search against NCBI “nr” database. Species were temporarily assigned by the best hits (*P* < 1e−5), and further confirmations were done by comparing the relative abundances of these species determined by metagenomic shotgun sequencing method and by 16S sequencing method. The OTU “New.ReferenceOTU131” represents *C. perfringens*, which is the most dominant OTU in some dogs, and OTU “HQ776819.1.1426” corresponds to *C. hiranonis*.

### Metagenomic sequencing and data analysis

Sequencing libraries were prepared using Illumina Nextera XT with 1 ng DNA extracted from canine stool collected at days 0, 14, and 42 from 18 out of the 20 diet-responsive dogs in the study. Sizing and quantification of libraries were carried out using a Tapestation 4200 (Agilent) and Qubit 3 (Thermo Fisher), respectively. Equimolar amounts of each library were pooled and sequenced on an Illumina NextSeq 500 instrument to produce 150 bp paired-end sequences. Sequencing adapters and low-quality reads were trimmed and filtered by Trimmomatic (v0.36) (leading:3 trailing:3 slidingwindow:4:15 minlen:36). High-quality reads were mapped to the canine reference genome (CanFam3.1), using Bowtie2 v2.3.4.1 (--very-sensitive), and aligned reads were removed using SamTools [81]. After host read filtering, each sample had a depth of at least 10 million paired-end reads (median depth = 35.8 million). Taxonomic annotation of each sample was generated using Metaphlan2 [49]. Functional and pathway composition was calculated with HUMAnN2 [82] using the UniRef50 database and UniPathway database with default settings. The abundance of each gene family in each species was estimated using HUMAnN2 and normalized with relative abundance. Bile salt hydrolase/choloylglycine hydrolase (BSH)-coding genes and genes in *bai* operon in microbiome were identified from the gene family search result of HUMAnN2 according the reference cluster IDs of these genes in UniRef50. Low abundance gene pathways with max abundance < 0.01% across all samples or present in < 25% samples were filtered out. Two-sided Wilcoxon signed-rank test was used for comparisons of UniPathway pathways at different timepoints (FDR < 0.05). Principal component analysis for pathways was performed by the R package factoextra. The identified *Clostridium* spp. and *Eubacterium* spp. were further searched for the existence of genes involved in secondary bile acid production (*bai* operon) using tBlastn against the reference genomes of these species in GenBank with the protein sequence of genes in 7α-dehydroxylation pathway (*baiG*, *baiB*, *baiA*, *baiF*, *baiCD*, *and baiE*) (*P* value ≤ 1e−5).

Metagenomic data from pediatric Crohn’s disease patients before and after exclusive enteral nutrition (EEN) have been described previously [23] and were downloaded from European Nucleotide Archive (ENA) (SRP057027). The same filtering steps and settings for the metagenomic data analysis above in this study were used for these datasets. After filtering out human reads, taxonomic annotation for each sample using Metaphlan2 showed the presence of *Clostridium*. Among them, *Clostridium scindens* has been well known for the secondary BA-producing ability. Paired reads with PCR duplicates removed by samtools [81] were aligned to the *C. scindens* reference genome (ASM15450v1, strain ATCC 35704) as well as strain VE202-05 (ASM47184v1) using bwa-mem (v0.7.17-r1188) [83] with default settings to estimate the relative abundances of bacteria among different samples (proportion of mapped reads in total reads). Wilcoxon’s sum rank test was used to test for significant differences in read mapping, and Spearman’s correlation was used to compare number of reads mapped with log-transformed fecal calprotectin (FCP) levels [23].

### Anaerobic culture and identification of bacterial isolates by whole genome sequencing

Rectal swabs freshly collected from dogs with active disease (day 0) and/or in remission at the end of the study (day 42) were transferred to an anaerobic chamber (97.5% nitrogen, 2.5% hydrogen; Coy Labs, Grass Lake, MI) within 1 h of collection. The tip of each swab was homogenized in 1 mL of pre-reduced PBS with 1% cysteine (PBSc). Serial dilutions made in PBSc (down to 10^−5^) were plated on brain-heart infusion (BHI), yeast casitone fatty acid with carbohydrate (YCFAC) [84], gut microbiota medium (GMM) [85], and De Man, Rogosa, and Sharpe (MRS) [86] agars (Anaerobe Systems, Morgan Hill, CA). After incubation at 37 °C for 1–3 days, single colonies were picked from plates and grown overnight in BHI, YCFAC, GMM, or MRS broth (Anaerobe Systems, Morgan Hill, CA). Overnight cultures were saved as glycerol stocks (25% glycerol) and frozen neat for DNA extraction. DNA was purified from bacterial isolates using the High Pure PCR template kit (Roche) and used for PCR with primers specific for the bacterial 16S rRNA gene, including 27F (5′-AGAGTTTGATCMTGGCTCAG-3′), 515F (5′-GTGCCAGCMGCCGCGGTAA-3′), and 1492R (5′-CGGTTACCTTGTTACGACTT-3′). PCR products were purified using QiaQuick PCR Purification kit (Qiagen) and Sanger sequenced, and sequences were assembled using Geneious software v11.1.5 (Biomatters, Inc.). The longest high-quality stretch of assembled sequence (at least 800 bp) was used for BLAST to find the closest match in GenBank. In addition, for selected *C. hiranonis*, *C. perfringens*, and *E. coli* isolates, 1 ng of DNA was used to construct sequencing libraries using Illumina Nextera XT. Libraries were sized and quantified as described above for metagenomic sequencing. Sourmash was used to detect contamination reads from metagenomic sequencing for the isolates [87]. For each sample, at least ~ 10 million, 150 bp single-end reads were generated using an Illumina NextSeq 500 instrument. Quality control steps were the same as the metagenomic analysis above. High-quality reads were mapped to the genome of *C. hiranonis* (ASM15605v1) using Stampy [88] (--substitutionrate = 0.1), which allows mapping of reads that are highly divergent from the reference genome. PCR duplicates were removed by Samtools. Coverage of genomic regions representing the *bai* operon was calculated for each isolate to show the existence of genes in 7α-dehydroxylation pathway.

### Metabolomics and in vitro bacterial growth inhibition assays

Bile acids were quantified in stool using a Waters Acquity uPLC System with a QDa single quadrupole mass detector and an autosampler as described previously [89]. Briefly, fecal samples were suspended in methanol (5 μL/mg stool), vortexed for 1 min, and centrifuged at 13,000*g* for 5 min. The supernatant was transferred to a new vial and analyzed on an Acquity uPLC with a Cortecs UPLC C-18+ 1.6 mm 2.1 × 50 mm column. All chemicals and reagents were mass spectrometry grade. The differential level analysis of bile acids was performed using the two-sided Wilcoxon signed-rank test. For the component below detection limit (< 1 nmol/g), the value 0.5 (half the limit of detection) was given for analysis. Canine isolates of *C. perfringens* (*n* = 3) and *E. coli* were revived from glycerol stocks in Modified Reinforced Clostridial Broth (MRCB, Fisher Scientific) or Luria broth (LB, Fisher Scientific), respectively, and grown overnight in the anaerobic chamber at 37 °C. Lithocholic and deoxycholic acids (Sigma) were dissolved in 100% ethanol (30 mg/mL). Growth inhibition by deoxycholic acid was determined by microbroth dilution and assessed by OD_630_ after overnight growth. Due to low solubility (< 1 mg/L), inhibition by lithocholic acid was assessed by counting colonies on agar plates with LCA (0, 0.1, 0.25, 0.5, 0.75, or 1 mg/mL and LB plates for *E. coli*, and 0, 0.01, 0.025, 0.05, 0.075, 0.01, 0.25, 0.50, 0.75, or 1 mg/mL and Columbia blood agar supplemented with 5% defibrinated sheep’s blood for *C. perfringens*) that were incubated anaerobically, at 37 °C for 24 (*E. coli*) or 48 h (*C. perfringens*). Growth curves (Fig. 4c, d) were run in 96-well plates using an Epoch2 plate reader (BioTek, Vermont, USA). Optical density measurements were taken at 600 nm every 15 min for 24 h. Wells containing 200 μL of the appropriate medium and bile acid levels were inoculated 1:100 (v/v) from standing overnight cultures. Technical replicates (*n* = 3) were run for bacterial strain under each condition. All procedures were conducted in an anaerobic chamber (Coy Labs, Michigan, USA).

### Mouse experiments

Female 7-week-old C57BL/6 J mice (specific pathogen-free) were purchased from Jackson Laboratories and maintained for 2–3 weeks in the animal facility at University of Pennsylvania prior to the to the start of dextran sulfate sodium (DSS) treatment. Animals were randomly assigned to groups (*n* = 5 mice per cage) at baseline, and drinking water was replaced with either filter-sterilized water (mock-treatment) or a filter-sterilized solution of 2.5% (w/v) DSS (relative molecular mass ~ 40,000; Sigma-Aldrich) in water. The mice treated with mock or DSS were orally gavaged *C. hiranonis* (1 × 10^8^ CFU/mouse, in anaerobic PBS) or anaerobic PBS (control) from days 0 to 4. *C. hiranonis* was grown overnight in MRCB, anaerobically, at 37 °C. Culture density was assessed via optical density (630 nm) and the required volume of culture was spun at 10,000 g for 15 min. The consumption of DSS water was monitored every day, and no significant difference was observed between the groups. Stool samples were collected at day -1 and day 3 for metabolomic analysis. Mice were euthanized on day 8, and colon tissues were collected. Distal colons were cut, fixed in formalin, embedded in paraffin, and stained with hematoxylin and eosin (H&E) or Periodic acid–Schiff (PAS). Histologic sections were evaluated by a board-certified veterinary pathologist (CB). Colitis was scored according to previously published criteria [90, 91]. Briefly, points were assigned to degree of mucosal/crypt loss (0: normal to 4: loss of all epithelium), lamina propria mononuclear infiltrate (0: normal to 3: marked increase), neutrophilic infiltrate (0: normal to 3: marked increase), epithelial hyperplasia (0: normal to 3: marked), and edema (0: normal to 3: severe and transmural).

For *E. coli* colonization experiments (Fig. 5k), C57BL/6 J mice were orally gavaged with kanamycin-resistant *E. coli* Nissle strain 1917 (1 × 10^9^ CFU/mouse) in LB media at day -3. Then, the mice were subjected to the same procedure as described above (treated with either DSS or mock; gavaged with *C. hiranonis* or anaerobic PBS). At day 8, colonic content was harvested, weighed, and plated on LB agar plates under kanamycin selection (100 μg/mL). *E. coli* colonies were counted and normalized by sample weight for each mouse.

## Additional files

Additional file 1: **Figure S1.** Detailed clinical design of the ‘ENTiCE’ canine chronic enteropathy study. **Figure S2.** Community structure of microbiomes in the dogs with CE and in the healthy dogs. **Figure S3.** Differentially abundant OTUs between healthy animals and CE animals. **Figure S4.** Microbiota community structure changes induced by diet therapy. **Figure S5.** OTU level (mean values) dynamics of microbiota structure for DR animals throughout the study. **Figure S6.** Concentrations of bile acids detected in fecal samples from diet-responsive dogs. **Figure S7.** Bile salt hydrolase (BSH) abundance in DR animals. **Figure S8.** Heatmap of the top 25 most abundant species across the samples in DR animals based on metagenomic data. **Figure S9.** Relative abundance of *C. hiranonis* in diet responsive dogs calculated from metagenomic data. **Figure S10.** Representative H&E staining sections of distal colon tissues at day 8 (40 x objective). **Figure S11.** Disease score-matched analysis. (PDF 5033 kb)

Additional file 2: **Table S1.** OTUs with differential abundances between samples of healthy dogs and dogs with CE at day 0 (Healthy vs CE day 0). **Table S2.** OTUs with differential abundances between day 0-samples of diet responsive dogs and non-diet responsive dogs. **Table S3.** Genera with differential abundances between samples of day 14 and day 0 (day 14 versus day 0) for diet responsive dogs. **Table S4.** OTUs with differential abundances between samples of day 14 and day 0 for diet responsive dogs (day 14 versus day 0). **Table S5.** OTUs with differential abundances between samples of day 14 and day 0 (day 14 versus day 0) for non-diet responsive dogs. **Table S6.** Differentially abundant pathways between day 14 and day 0 after diet treatment from metagenomic analysis for diet-responsive dogs (fold-change > 1.5 and *P*-value < 0.05). **Table S7.** Comparisons of bile acids in samples at different timepoints for diet responsive dogs. **Table S8.** Spearman correlations between abundance of OTUs and concentration of Bile acids in diet responsive dogs. **Table S9.** Spearman correlations between bile acids and bile salt hydolases (BSHs) in diet-responsive dogs (P < 0.05). (XLSX 135 kb)

## Data Availability

Raw 16S rRNA gene sequences and metagenomic sequences for all samples used in this study have been deposited in the Sequence Read Archive (SRA) under project accession no. PRJNA515316. Processed 16S data and clinical metadata for the ENTiCE study are also available on microbiomeDB.org [92]. Original R scripts, metadata, and OTU table are available on Github at https://github.com/dpbisme/Wang_ENTiCE_study, and have been archived on Zenodo (DOI: 10.5281/zenodo.3379096).

## Abbreviations

ENTiCE: Effect of Nutritional Therapy on Microbiome in Canine Enteropathy
PLEASE: Pediatric Longitudinal Study of Elemental Diet and Stool Microbiome Composition
BA: Bile acid
BSH: Bile salt hydrolase
CCE: Canine chronic enteropathy
CCECAI: Canine Chronic Enteropathy Clinical Activity Index
CE: Chronic enteropathy
DCA: Deoxycholic acid
DR: Diet-responsive
EEN: Exclusive enteral nutrition
ENA: European Nucleotide Archive
FDR: False discovery ratio
FMT: Fecal microbiota transplant
FRE: Food-responsive enteropathy
IBD: Human inflammatory bowel disease
LCA: Lithocholic acid
NDR: Non-diet-responsive
OTU: Operational taxonomic unit
PEN: Partial enteral nutrition
QIIME: Quantitative Insights into Microbial Ecology
SRA: Sequence Read Archive
