# Supplementary material for: Diet-induced remission in chronic enteropathy is associated with altered microbial community structure and synthesis of secondary bile acids
Source: Microbiome. 2019 Aug 31;7:126. doi: 10.1186/s40168-019-0740-4 (PMC6717631; doi:10.1186/s40168-019-0740-4)
Supplement: Supplementary file 1 — Figure S1. Detailed clinical design of the ‘ENTiCE’ canine chronic enteropathy study. Figure S2. Community structure of microbiomes in the dogs with CE and in the healthy dogs. Figure S3. Differentially abundant OTUs between healthy animals and CE animals. Figure S4. Microbiota community structure changes induced by diet therapy. Figure S5. OTU level (mean values) dynamics of microbiota structure for DR animals throughout the study. Figure S6. Concentrations of bile acids detected in fecal samples from diet-responsive dogs. Figure S7. Bile salt hydrolase (BSH) abundance in DR animals. Figure S8. Heatmap of the top 25 most abundant species across the samples in DR animals based on metagenomic data. Figure S9. Relative abundance of C. hiranonis in diet responsive dogs calculated from metagenomic data. Figure S10. Representative H&E staining sections of distal colon tissues at day 8 (40 x objective). Figure S11. Disease score-matched analysis. (PDF 5033 kb) [file 40168_2019_740_MOESM1_ESM.pdf]

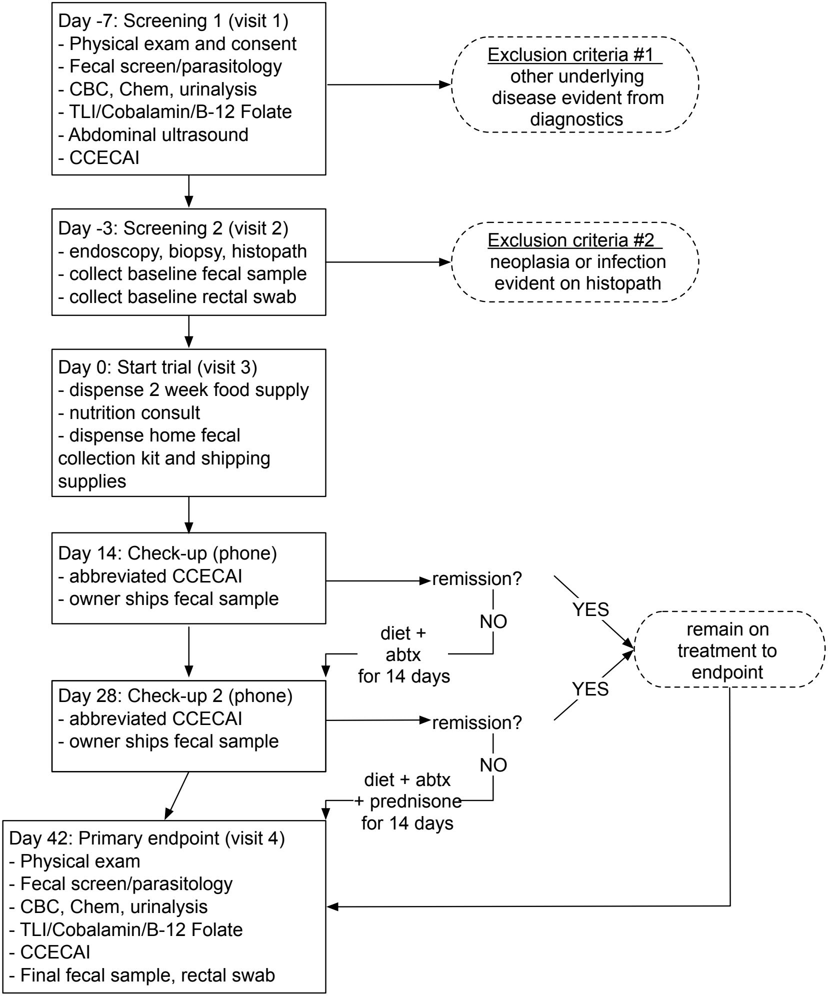

Fig. S1. Detailed clinical design for the 'ENTiCE' canine chronic enteropathy study.

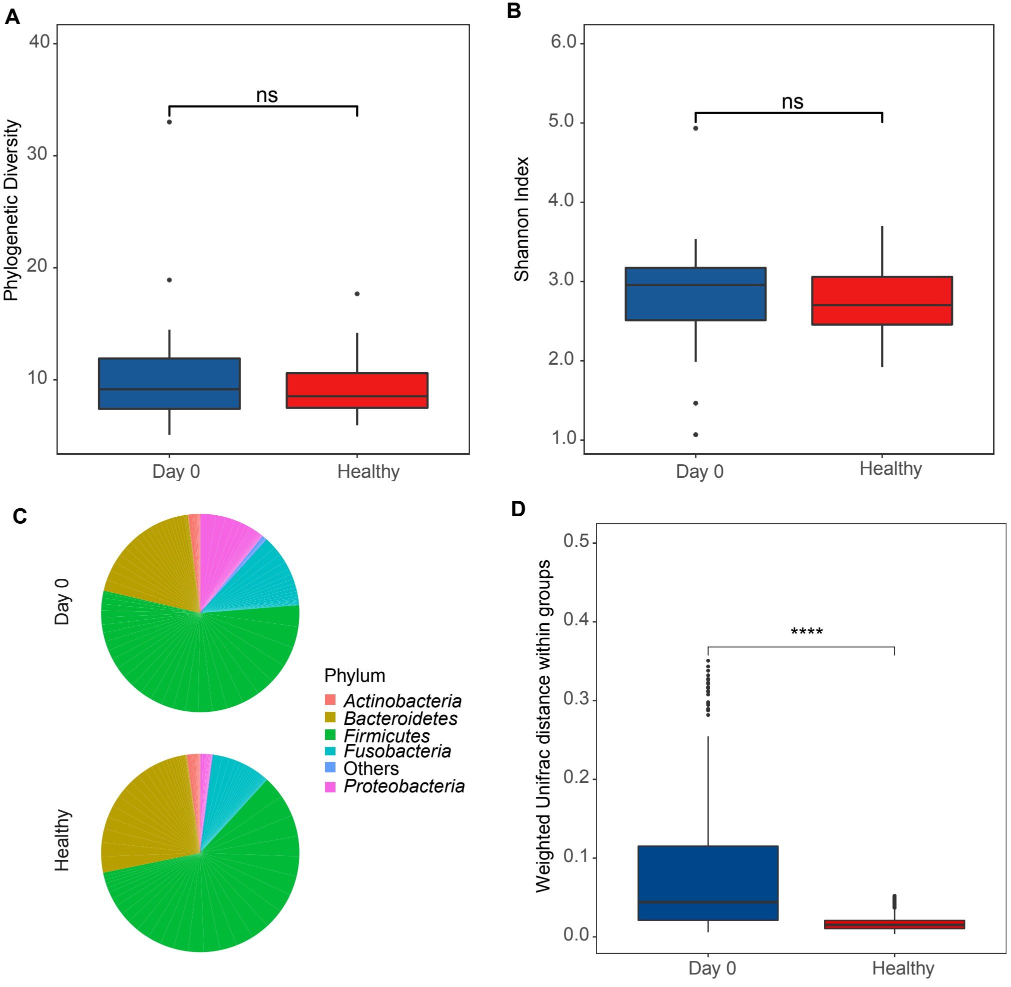

Fig. S2. Community structures of microbiomes in the dogs with CE and in the healthy dogs. Faith's phylogenetic diversity (A) and Shannon index (B) were compared between the samples from the dogs with CE (day 0) and the samples from healthy dogs. (C)The ratios of microbiota compositions at a phylum level. (D) Weighted Unifrac distances within the microbiomes of the dogs with CE or within those of the healthy dogs. ns = not significant, ****$p < 0.0001$ using two-sided Wilcoxon rank sum test.

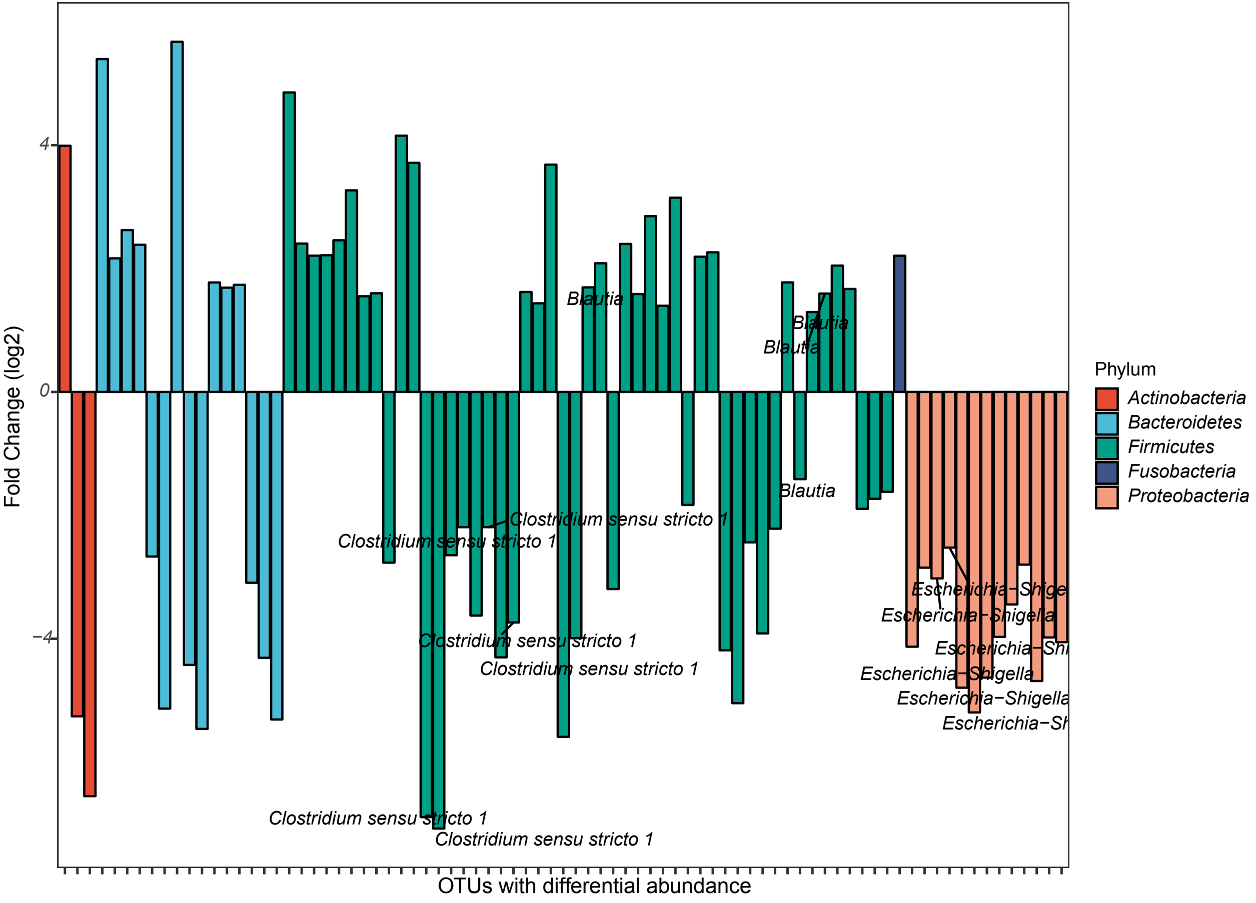

Fig. S3. Differentially abundant OTUs between healthy animals and CE animals. OTUs with differential abundances were identified using DESeq2 (log2(Fold-Change) >1 and P< 0.05). OTUs from *Fusobacteria*, *Firmicutes*, *Actinobacteria*, *Proteobacteria,* and *Bacteroidetes* are shown.

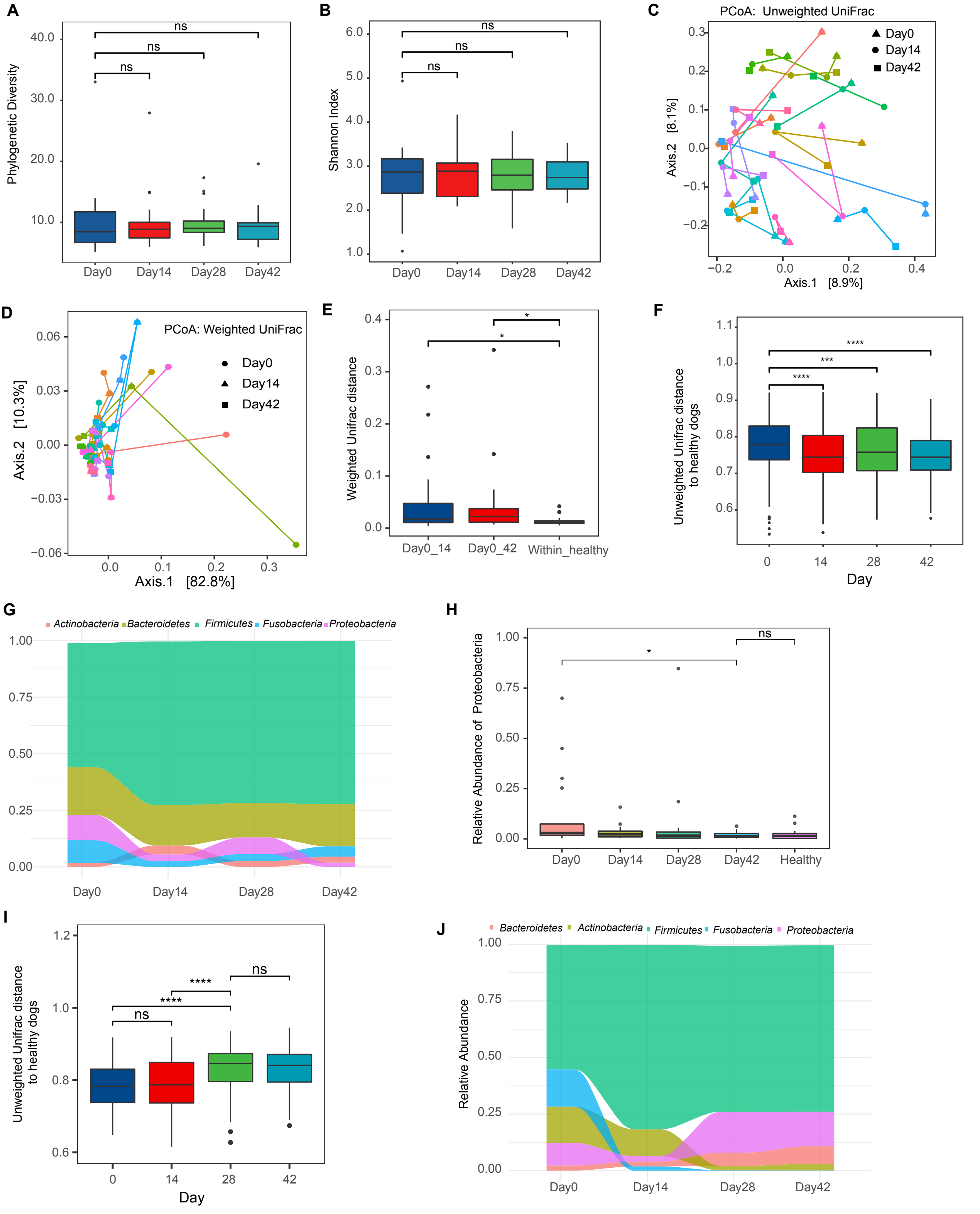

Fig. S4. Microbiota community structure changes induced by diet therapy. (A) Faith's phylogenetic diversity for DR animals. (B) Shannon index for DR animals. (C) Principal coordinate Analysis (PCoA) based on unweighted Unifrac distance or (D) weighted UniFrac distance of the microbiomes from DR animals. (E) Weighted UniFrac distance of microbiomes between samples at different timepoints for each dog in DR group (Day0_Day14 and Day0_Day42) and between animals within healthy control (within_healthy). 'Day0_Day14' and 'Day0_Day42' represent the shift of microbiome measured by weighted UniFrac distance from day 0 to day 14, or day 0 to day 42, respectively, for each dog in DR group. 'Within_healthy' represents the heterogeneity of microbiome within healthy control group. (F) Unweighted Unifrac distance to healthy dogs for DR animals. (G) Stream plot showing phylum level (mean values) dynamics of microbiota structure for DR animals throughout the study. (H) Relative Abundance of the phylum *Proteobacteria* for DR animals after diet and for healthy animals. (I) Unweighted Unifrac distance to healthy dogs for NDR animals. (J) Stream plot showing phylum level (mean values) dynamics of microbiota structure for NDR animals throughout the study. ns = not significant, *p < 0.05, ***p < 0.001, ****p < 0.0001 using two-sided Wilcoxon rank sum test.

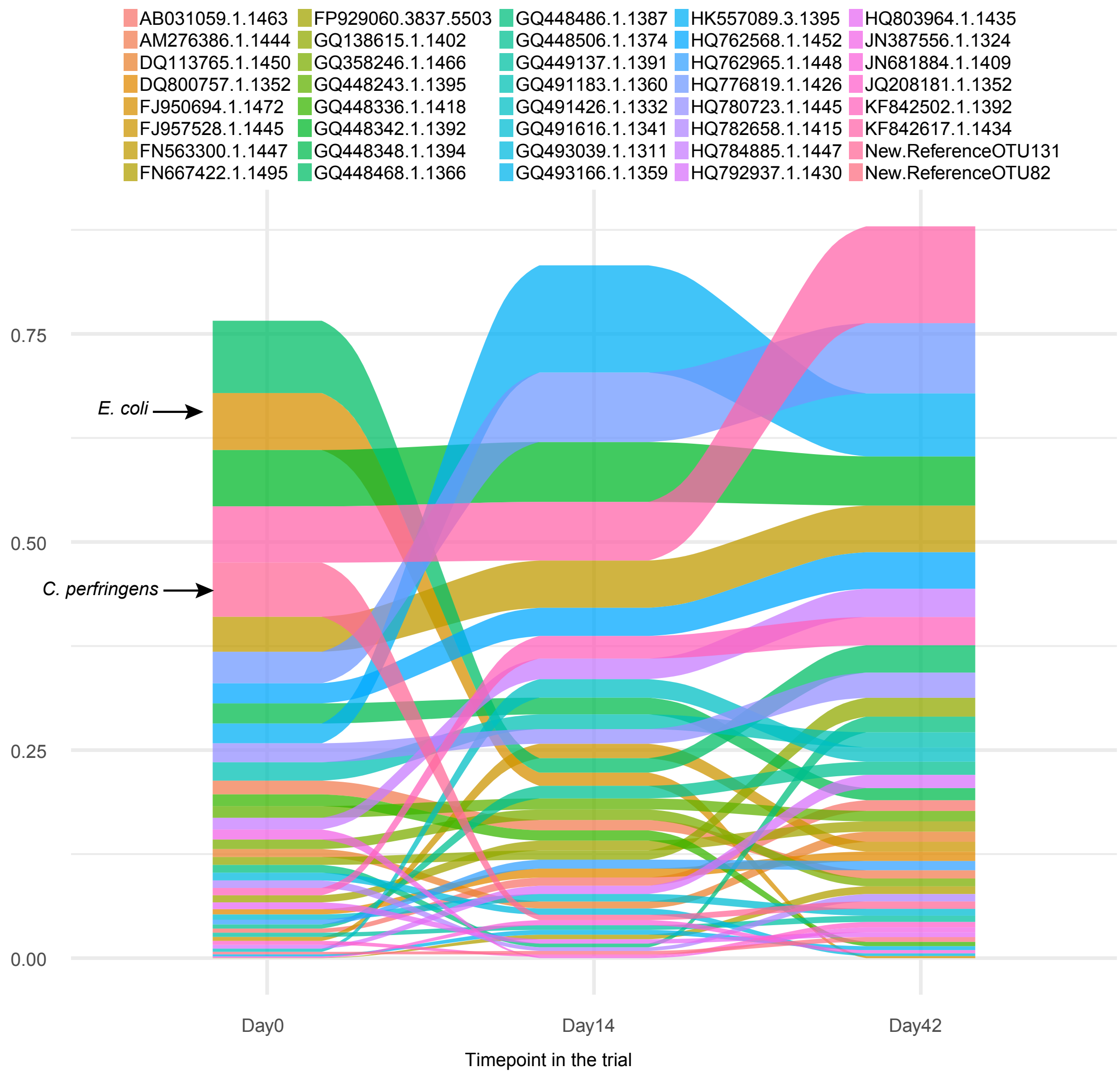

Fig. S5. OTU level (mean values) dynamics of microbiota structure for DR animals throughout the study. Stream plot showing the changes in the relative abundance of the top 40 most abundant OTUs across the timepoints in the ENTiCE study. *E. coli* and *C. perfringens* are indicated with arrows.

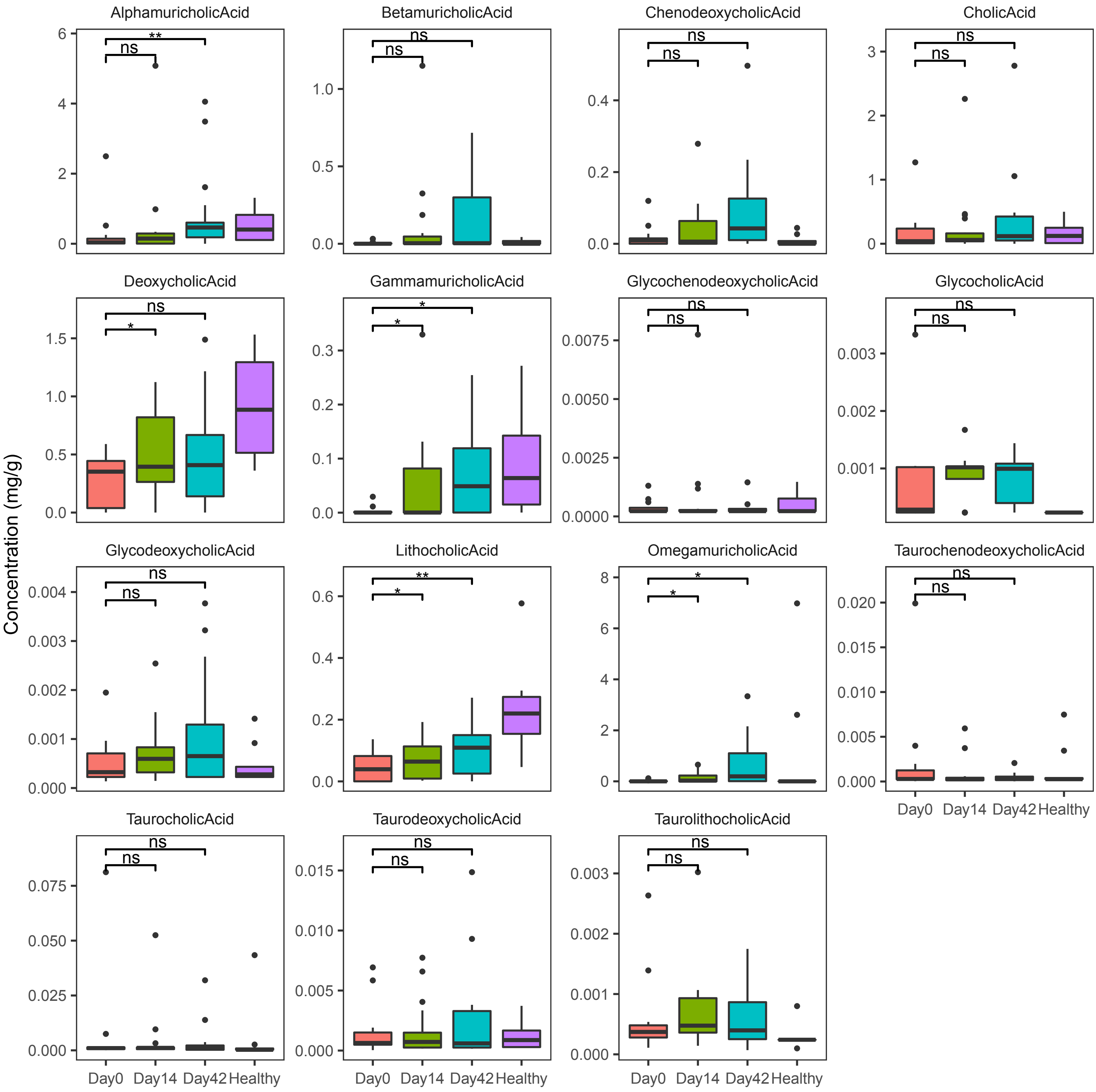

Fig. S6. Concentrations of bile acids detected in fecal samples from diet-responsive dogs. Concentrations (mg/g stool sample) were converted from nmol/g with molecular weights. ns = not significant, *p < 0.05, **p < 0.01 using two-sided Wilcoxon signed-rank test.

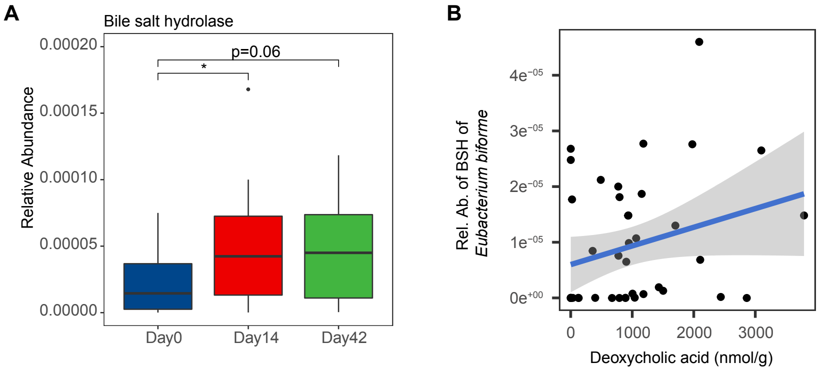

Fig. S7. Bile salt hydrolase (BSH) abundance in DR animals. (A) The relative abundances of BSH gene in DR animals based on metagenomic data. The relative abundance for genes with homology to UniRef 50 reference cluster UniRef50_Q06115 is shown. (B) Correlation between the relative abundance (Rel. Ab.) of BSH in *Eubacterium biforme* and deoxycholic acid concentration in stool samples for DR animals. The species-level abundance of BSH was estimated using HUMAnN2. ns = not significant, *p < 0.05, using two-sided Wilcoxon rank sum test.

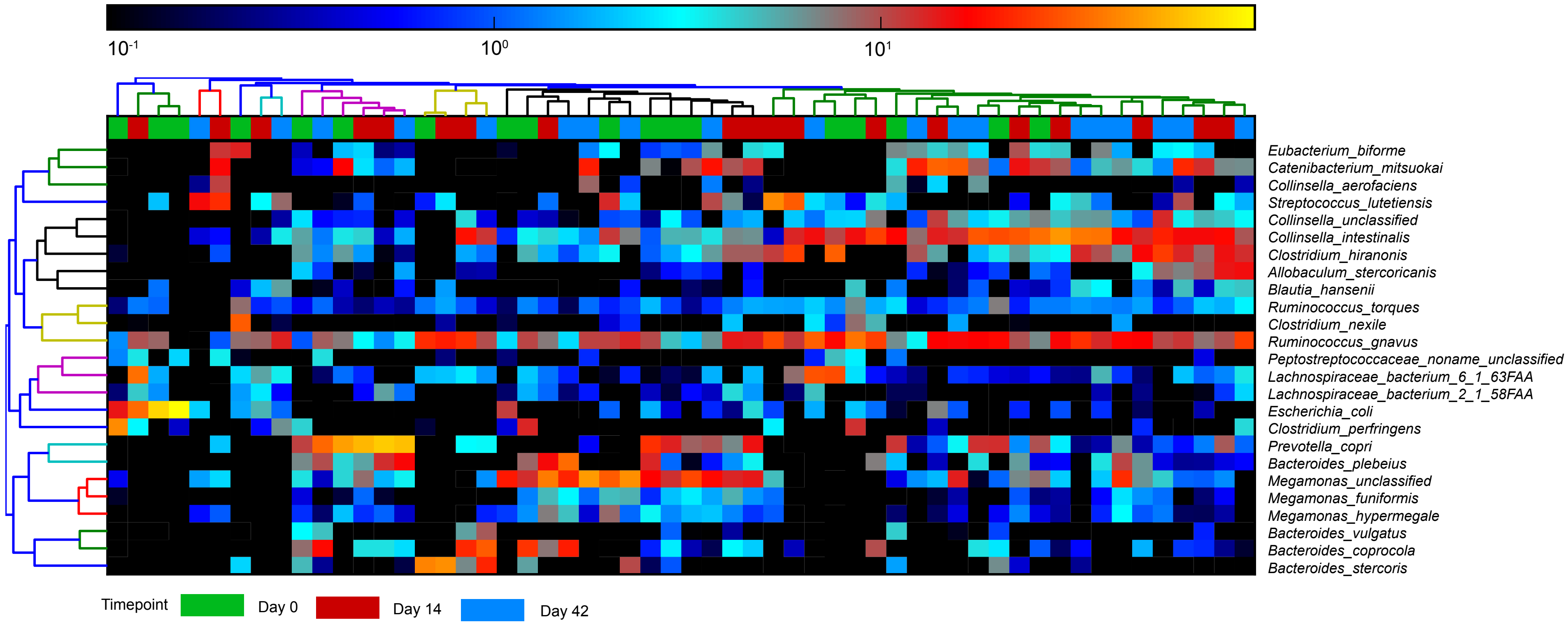

Fig. S8. Heatmap of the top 25 most abundant species across the samples of DR animals based on metagenomic data. The abundances were log-scaled. 'Braycurtis' method implemented in Hclust2 was used to measure the distance between samples. 'Correlation' method was used for measuring distance between species.

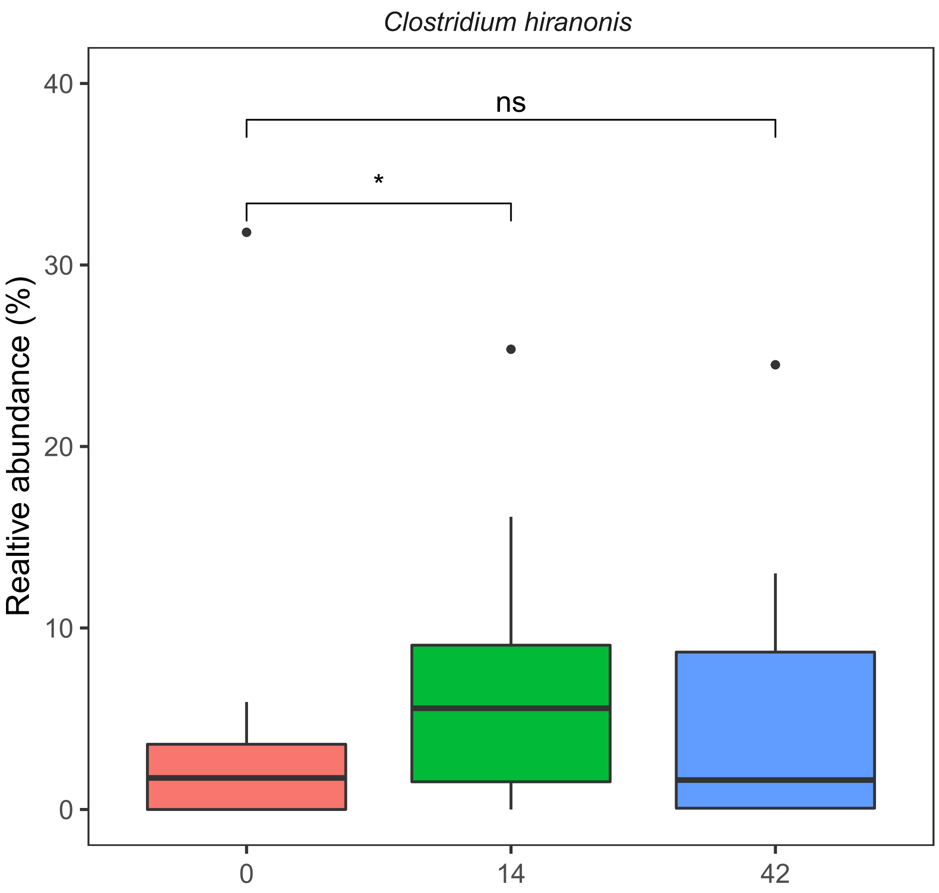

Fig. S9. Relative abundance of *C. hiranonis* in diet responsive dogs calculated from metagenomic data. ns = not significant, *p < 0.05, using two-sided Wilcoxon signed-rank test.

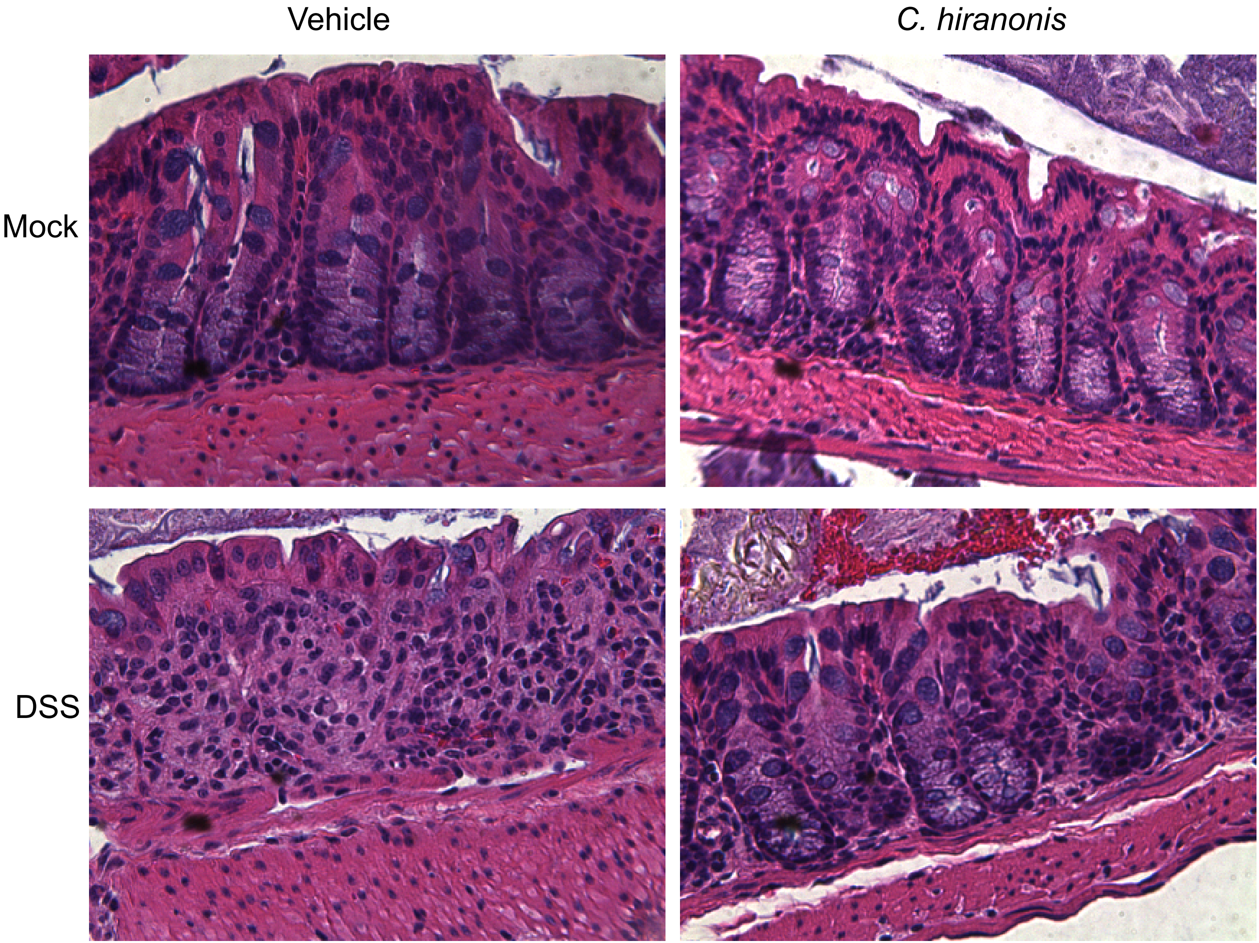

Fig. S10. Representative H&E staining sections of distal colon tissues at day 8 (40 x objective).

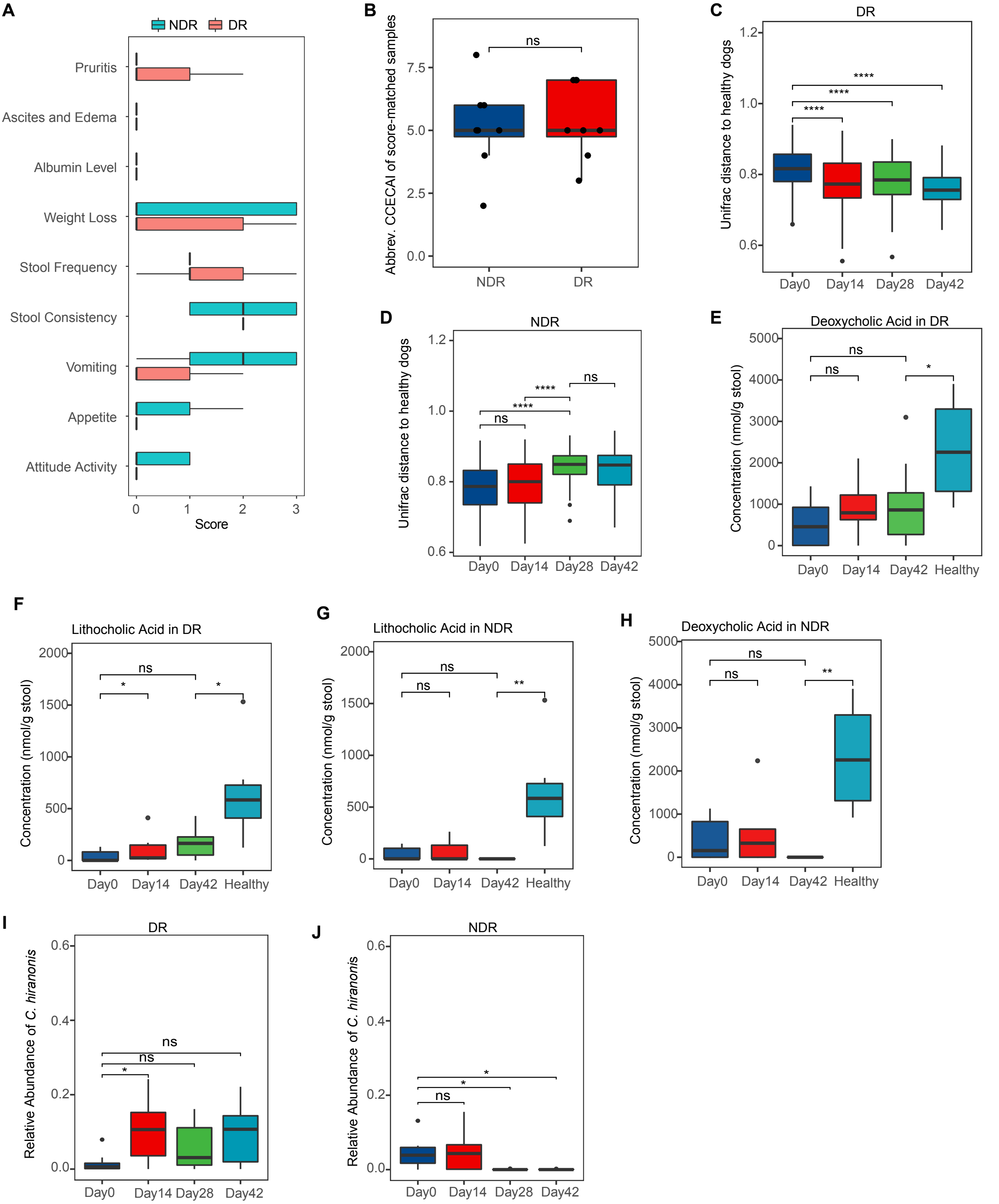

Fig. S11. Disease score-matched analysis. (A) Comparison of CCECAI component scores between DR and NDR animals. (B) CCECAI distribution for animals used in disease scores-matched analysis. (C) Unweighted UniFrac distance of DR group to healthy group. (D) Unweighted UniFrac distance of NDR group to healthy group. Deoxycholic acid levels for (E) DR animals and (H) NDR animals, and lithocholic acid levels for (F) DR animals and (G) NDR animals are shown. (I) Relative abundance of C. hiranonis in DR animals and (J) NDR animals. ns = not significant, *p < 0.05, **p < 0.01,****p < 0.0001 using two-sided Wilcoxon rank sum test for panels B-H or two-sided Wilcoxon signed rank (paired) test for panels I and J.
